# Pwp2 mediates UTP-B assembly via two structurally independent domains

**DOI:** 10.1038/s41598-017-03034-y

**Published:** 2017-06-09

**Authors:** Fanny Boissier, Christina Maria Schmidt, Jan Linnemann, Sébastien Fribourg, Jorge Perez-Fernandez

**Affiliations:** 1 Université de Bordeaux, INSERM U1212, CNRS 5320 Bordeaux, France; 2 Universität Regensburg, Biochemie-Zentrum Regensburg (BZR), Lehrstuhl Biochemie III, D-93053 Regensburg, Germany

## Abstract

The SSU processome constitutes a large ribonucleoprotein complex involved in the early steps of ribosome biogenesis. UTP-B is one of the first multi-subunit protein complexes that associates with the pre-ribosomal RNA to form the SSU processome. To understand the molecular basis of the hierarchical assembly of the SSU-processome, we have undergone a structural and functional analysis of the UTP-B subunit Pwp2p. We show that Pwp2p is required for the proper assembly of UTP-B and for a productive association of UTP-B with pre-rRNA. These two functions are mediated by two distinct structural domains. The N-terminal domain of Pwp2p folds into a tandem WD-repeat (tWD) that associates with Utp21p, Utp18p, and Utp6p to form a core complex. The CTDs of Pwp2p and Utp21p mediate the assembly of the heterodimer Utp12p:Utp13p that is required for the stable incorporation of the UTP-B complex in the SSU processome. Finally, we provide evidence suggesting a role of UTP-B as a platform for the binding of assembly factors during the maturation of 20S rRNA precursors.

## Introduction

Eukaryotic ribosome biogenesis is a complex process that involves the coordinated synthesis, processing and folding of the four ribosomal RNAs (rRNAs) along with the stable assembly of 79 ribosomal proteins (RPs). In addition, more than 200 assembly factors (AFs) associate transiently with pre-ribosomal particles to guide ribosome maturation. Biogenesis is also spatially controlled, commencing in the nucleolus and finishing in the cytoplasm, with a mandatory transition through the nuclear pore complex. AFs include proteins and snoRNAs, which participate in the processing and folding of rRNA, the chemical modification of specific rRNA nucleobases and the binding of RPs. The association of different sets of AFs during the assembly of ribosomes are clear landmarks of early, middle and late assembly events. The early events of ribosome biogenesis include the synthesis of a single, large transcript called 35S in the yeast *Saccharomyces cerevisiae*. In these yeast cells, around 50 AFs and several snoRNAs, such as U3, U14 and snR30, associate co-transcriptionally with the 35S pre-RNA to form the first pre-ribosomal particle known as 90S or the SSU-processome^1–4^. Due to their association with the U3 snoRNA, some of those AFs have been renamed as Utps (U three proteins)^1^. Moreover, some of these Utps have been shown to remain associated independently of pre-ribosomal particles into complexes known as UTP-A, UTP-B, and UTP-C^5^. The association of UTP complexes with the pre-ribosomal RNA appears to be a hierarchical process^6^. In this regard, binding of the UTP-B complex to the pre-rRNA requires the previous binding of the UTP-A complex. The binding of both UTP-A and UTP-B complexes constitutes a mandatory step required for the subsequent recruitment of other AFs such as the Mpp10 complex, Utp20p, Rrp5p, Rrp36p, and the Bms1p-Rcl1p heterodimer^7–9^. Although a large set of AFs participating in different steps of pre-20S maturation, including the Mpp10 complex, the Bms1p-Rcl1p dimer, and the U3 snoRNP among others associate with specific pre-rRNA domains^10, 11^, it cannot explain the hierarchical relationships between AFs during the earlier events of ribosome biogenesis. Moreover, the recent resolution of SSU-processome related structures identify the position of several AFs in the pre-rRNA^12–15^, but the protein-protein interactions related with the recruitment of AFs to the SSU-processome remains unknown.

The UTP-B complex consists of Utp6p, Utp12p/Dip2p, Utp13p, Utp18p, Utp21p, and Pwp2p/Utp1p^5^. The hexameric organization of the UTP-B complex is compatible with an observed aggregated mass of around 600 kDa^16^, assuming one copy per subunit. A network map of interactions between the UTP-B subunits has emerged from two-hybrid experiments, mass spectrometry analysis of cross-linked proteins and reconstitution assays in heterologous systems^16–18^. Utp18p and Utp6p form a dimer that requires the association of Utp21p to recruit Pwp2p to form the core complex of UTP-B^16^, most probably through direct interactions between Utp21p and Pwp2p^18^. The incorporation of Pwp2p is essential for the subsequent association of the Utp12p-Utp13p dimer. Recently, several laboratories have reported cryo-EM structures at various resolution of pre-ribosomal particles that include among many other AFs, the UTP-B complex^13, 15, 19^.

Apart from this molecular description, the role of UTP complexes in early ribosome biogenesis is not totally understood. Although most of the UTP-A components have been related to a transcriptional optimization of the rDNA by the RNA polymerase I^20^, this aspect is still under debate^21^. Furthermore, it has been suggested that the UTP complexes would play an RNA chaperone function during the assembly of the SSU processome^12, 13^. To understand the role of the individual subunits and the intact UTP complex, it is interesting to solve the individual structures of the subunits and any higher order assemblies up to the full complex. We report the structure of the N-terminal domain of Pwp2p, which exhibits a prototypical tandem WD40 repeat (tWD). We also show that the C-terminal domain (CTD) of Pwp2p is required for the stable formation of the core complex and for the assembly of Utp12p and Utp13p into a complete UTP-B. Formation of the full UTP-B complex seems to be a key aspect of its stable association with pre-rRNA, which in turn is essential for the formation of the SSU-processome. Additionally, we show that UTP-B is involved not only in the early steps of pre-rRNA maturation but also in the late steps of nuclear maturation of 20S pre-rRNA.

## Results

### Pwp2p is essential for UTP-B and SSU-processome formation

**Figure 1:**
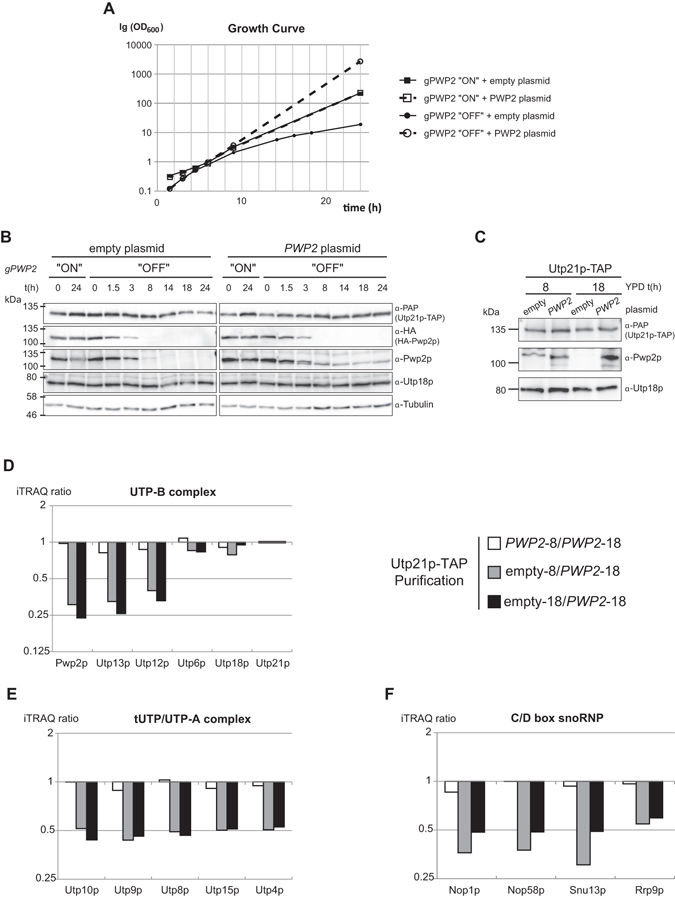
Pwp2p depletion affects UTP-B composition and its association with other SSU processome complexes. (**A**) Growth curves of the yeast strain yCMS3-1a harbouring either a plasmid containing a wild-type copy of *PWP2* (light) or only the plasmid backbone (“empty”, in dark) were cultivated in YPG to overexpress the endogenous Pwp2p (“ON”) or in YPD to shut down the expression of endogenous Pwp2p (“OFF”). (**B**) Culture aliquots equivalent to five optical units were collected at different time points during the time course. Proteins were extracted by the TCA-urea method (see Methods) and equal volumes from the cell extracts were analysed by western blot using antibodies against Protein A (anti-PAP), HA, Utp21p, Utp18p, Pwp2p, and Tub1p. (**C**) Yeast cells from the strain yCMS3-1a harbouring the *PWP2* containing plasmid (*PWP2*) or the plasmid backbone (empty) were cultivated in YPD. After 8 h or 18 h, cells were harvested and Utp21p-TAP was immuno-purified. Ten percent of the elution sample was then analysed by western blot using antibodies against Protein A (anti-PAP), Pwp2p, or Utp18p antibodies. (**D–F**) Utp21p-TAP-purified proteins were identified and quantitated by mass spectrometry. Isobaric labelling of peptides (iTRAQ, see Methods) was used to compare the protein levels in wild-type and Pwp2p-deleted strains. The bar graphs represent the ratios for the indicated purifications represented in logarithmic scale. The average ratios for the identified proteins (cut-off set for at least two peptides) in three independent experimental replicates is shown for UTP-B subunits (**D**), tUTP/UTP-A subunits (**E**) and C/D box snoRNP (**F**).

In order to characterize the essential function of Pwp2p mutants, we first investigated the effects of Pwp2p depletion on the protein expression  levels of the other UTP-B components. We used the yeast strain YCMS3-1a in which the genomic copy of *PWP2* is expressed, with an N-terminal HA tag, under the control of a galactose-inducible and glucose-repressed promoter. In addition, endogenous *UTP21* is expressed under its natural promoter and C-terminal tagged with the TAP-tagged coding sequence^22^. This strain was transformed with either an empty control plasmid or a plasmid expressing a wild-type version of *PWP2* under its natural regulatory sequences. Both strains were cultivated on YPD medium in order to deplete the genomic-encoded Pwp2p. Because Pwp2 is an essential protein, cell growth was observed only in strains bearing a functional *PWP2* (*PWP2* plasmid) (Fig. 1A). In contrast, the growth of cells where Pwp2p was depleted (empty plasmid) started to decline 8 h post Pwp2p depletion (Fig. 1A). In agreement with the *in vivo* data, the un-tagged Pwp2p but not the endogenous Pwp2p-HA was detectable by WB after 8 h of growth in glucose containing medium (Fig. 1B second panel from the top). Furthermore, the protein levels of Utp21 and Utp18 remained unchanged in the absence of Pwp2p (Fig. 1B top and third panels).

In order to analyse the effects of Pwp2p depletion on the association of Utp21p with other proteins, we induced the depletion of the genomic-encoded Pwp2p for 8 h and 18 h in the presence and in the absence of the plasmid-encoded copy of *PWP2*. Both time points delimit a timeframe in which depletion of Pwp2p does not cause a massive decrease in the protein levels of Utp21p and Utp18p. Afterwards, Pwp2p depleted and non-depleted cells were lysed and Utp21p-TAP associated complexes were affinity purified as described (see Methods). First, immuno-purified proteins were analysed by WB. According to the depletion of Pwp2p, our results showed a strong decrease in Pwp2p in the Utp21p-TAP purification at 8 h and 18 h after the shift to glucose-containing media (Fig. 1C). In contrast, co-purification of Utp21p with Utp18p was not affected by the absence of Pwp2p (Fig. 1C). Second, in order to analyse the proteome associated with Utp21p-TAP, we performed in solution semi-quantitative mass spectrometry (q-MS) using the iTRAQ labelling method^23^. Obtained data was normalized against the average amount of Utp21p-TAP peptides. According to WB analysis, the depletion of Pwp2p correlated with a strong decrease in Pwp2p associated with Utp21p 8 h after the glucose shift and continued to decrease 18 h after Pwp2p depletion started. Moreover, the impaired association of Utp21p with Pwp2p correlated with a strong reduction of the association of Utp12p and Utp13p with Utp21p (Fig. 1D). In contrast, co-purification of Utp21p with either Utp18p or Utp6p was not affected (Fig. 1D). Altogether, our results point to a Pwp2p-dependent assembly of Utp12p and Utp13p to Utp21p and a Pwp2p-independent assembly of Utp21p with Utp6p and Utp18p. Thus, Pwp2p is probably bridging the interaction between the Utp12p-Utp13p dimer and the core complex of UTP-B.

The extended q-MS analysis of other SSU-processome factors, such as the tUTP/UTP-A (Fig. 1E) and the U3snoRNP (Fig. 1F) components, reveals a reduced association of Utp21p with these complexes in the absence of Pwp2p. Furthermore, a detailed analysis of other SSU-processome components showed a decreased association of Utp21p with most of them (Supplementary Figure 1). Nevertheless, a couple of proteins, such as Nop6p and Mrd1p, did not show Pwp2p dependency in their association with Utp21p (Supplementary Figure 1).

Altogether, the association of Pwp2p with the heterotrimer Utp21p-Utp6p-Utp18p is a key step in the further association of Utp12p and Utp13p. Furthermore, either the association of the Utp12p:Utp13p dimer or the assembly of Pwp2p into the core complex is required for the association of Utp21p with other SSU-processome proteins. Finally, no major difference was observed between the results obtained at 8 h and 18 h after Pwp2p depletion had started; therefore, intermediate depletion time points were used in the following experiments.

### Overall structure of Pwp2p tandem WD-repeat domains and surface properties

In order to gain insights into the structure and domain definition of Pwp2p, we aimed to solve its structure. The WD-repeat-containing region of Pwp2p (residues 1–716) was subcloned, produced and purified as described in the Methods section. The purified protein was crystallized, and X-ray diffraction data were collected up to 2.54 Å resolution. The structure was solved by molecular replacement using the BALBES server^24^ that identified the PDB 2PBI (chain B, residues 55 to 351) as a good search model. Each tandem WD-repeat was placed individually by an iterative search to give an initial molecular replacement solution that was further refined with PHENIX^25^. The model was manually improved by sequential building and refinement iterations with PHENIX and Buster^25, 26^. The final model was refined to an Rfree of 23.9% (Table 1) (Methods).

**Table 1:** Data collection and refinement statistics.

	Pwp2 (1–716)
Wavelength (Å)	0.97857
Resolution range (Å)	19.95–2.54 (2.68–2.54)
Space group	*P*2_1_2_1_2_1_
Unit cell	70.8 76.04 191.35
Total reflections	304,721 (45,097)
Unique reflections	34,863 (4,990)
Multiplicity	8.7 (9.0)
Completeness (%)	99.8 (100.0)
Mean I/sigma(I)	12 (1.2)
Wilson B-factor (Å^2^)	76.9
R-pim (%)	0.046(0.651)
CC1/2 Mn(I) half-set correlation CC(1/2)	0.998 (0.536)
Reflections used for R-free	1,729
R-work (%)	0.192
R-free (%)	0.239
Number of non-hydrogen atoms	4,834 (iso) 4,785 (aniso)
Protein residues	707
RMS (bonds)(°)	0.009
RMS (angles)(Å)	1.304
Ramachandran favoured (%)	93.97
allowed (%)	5.70
outliers (%)	0.33
Clashscore	9.72
Average B-factor (Å^2^)	43.81
PDB	5I2T

**Figure 2:**
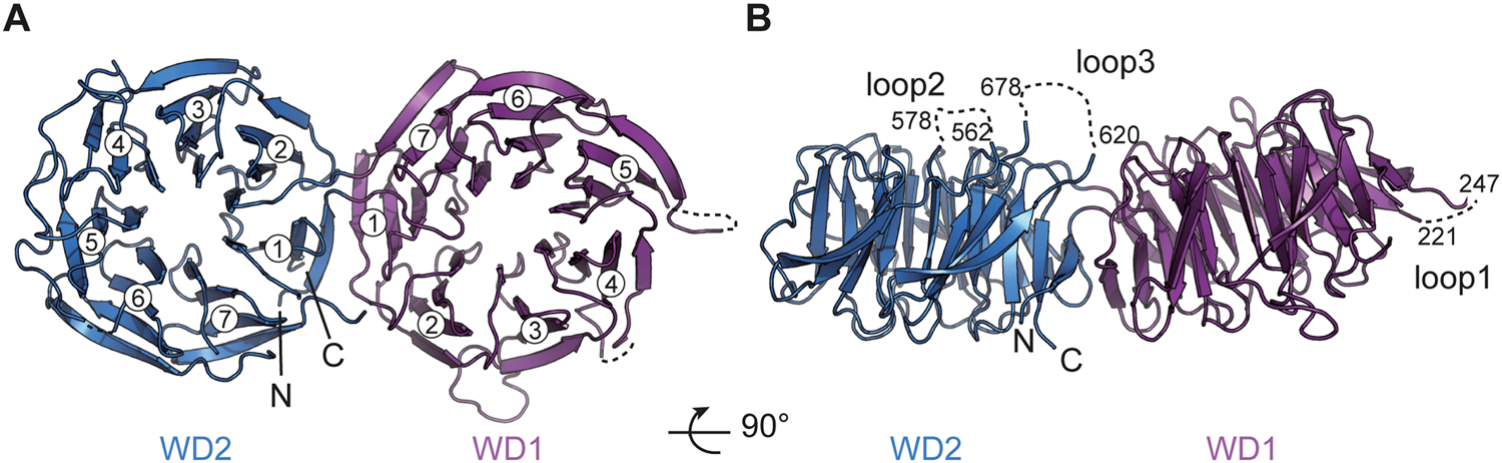
Overall structure of Pwp2p tandem WD-repeat domain. (**A**) The first WD-repeat domain (violet) is composed of residues 12–343, whereas WD2 (blue) is composed of residues 4–11 and 344–707. Blade numbers are indicated (**B**) Disordered loops are shown as dotted lines. The two panels are related to each other by a rotation of 90° along the x-axis. The N- and C-termini are labelled.

Over the 716 residues of the construct, the final model comprises 608 residues (from residue 4 to 707). Three disordered loops comprising residues 222–246 (Loop1), 561–576 (Loop2), and 621–677 (Loop3) could not be modelled into the electron density map and are not included into the final model (PDB 5I2T) (Fig. 2 and S2). Pwp2p (1–716) contains two canonical WD40 domains, WD1 and WD2. Each WD domain is a seven-bladed β-propeller composed of four anti-parallel β-strands. The topology follows the same rule as described earlier for other tandem WD repeats^27–29^. The first WD-repeat domain (WD1) is composed of residues 12–343. The second WD-repeat domain (WD2) is composed of residues 4–11 and 344–706 (Fig. 2). The connection between the two WD domains is ensured by residues 12–14 and 342–346 in addition to the external β-strands of blade 1 of each WD domain, and the loop connecting blade 1 and blade 2. The two WD domains form an angle of approximately 30°. WD1 and WD2 superimpose with an r.m.s.d. of 2.27 Å over 244 Cα carbons.

The UTP-B complex is composed of proteins containing predominantly WD repeats domains. WD repeats are scaffolds known to trigger various functions, including protein-protein interactions and nucleic acid recognition^28^. Given that UTP-B subunits are conserved from yeast to mammals, it is likely that conserved surface areas relate to regions that mediate essential protein-protein or other biomolecular interactions. In order to identify key determinants of Pwp2p function, we plotted the invariant residues at the surface of Pwp2p tandem WD-repeat structure using the CONSURF server^30^ and the alignment of orthologous Pwp2p sequences covering yeast to mammals (Supplementary Figures 2 and 3). Several patches of conserved residues were identified, some of which were spanning an extended area over WD1 and WD2 (Supplementary Figure 2). This area is located on the “side” (thinner portion) of the WD repeats, and it includes the invariant residues E335, K340, Q342, K368, R418, and R420. Electrostatic potential surface analysis of this area reveals a mixed composition of positively and negatively charged residues (Supplementary Figure 2C and D). From an electrostatic point of view, the other faces of Pwp2p display strongly charged areas Supplementary Figure 2E–H). Such a representation does not take into account the non-modelled loops. As mentioned earlier, approximately 100 residues could not be built into the final model. A quick inspection of the sequence alignments reveals that Loop1 (residues 221–247) is poorly conserved across species while Loop2 (residues 562–578) and Loop3 (residues 620–678) are conserved, and they likely comprise residues supporting the function (Supplementary Figure 3). Similarly, Pwp2p-CTD (not included in the crystallized construct) is also highly conserved.

### *In vivo* analysis of surface-exposed invariant amino acids of Pwp2p

To analyse the biological role of the different conserved and surface-exposed residues in Pwp2p, point mutants were generated. To analyse the phenotypes, we used the yeast strain where the endogenous *PWP2* was expressed under the control of a GAL promoter. As previously shown, depletion of Pwp2p expressed from the genomic copy of *PWP2* had a deleterious effect in the absence of a functional *PWP2* copy (Supplementary Figure 4A and B). Growth of yeast strains containing the *pwp2*-mutants E335R, K368E, R418E/R420E, E473R, and K613E/R614E was analysed at different temperatures after depletion of endogenous Pwp2p. The *pwp2* mutants E335R, K368E, R418E/R420E, and E473R did not show any growth defect at any temperature (Supplementary Figure 4A and B). In contrast, the expression of *pwp2-K613E/R614E* showed a mild growth defect at 30 °C, which increased slightly at 37 °C (Supplementary Figure 4B and D). In addition, a mutant lacking the region comprised of residues F693–A738 and coding for the two last β-sheets of the WD2 domain was generated by the deletion of a DNA region situated between two *Nsi*I restriction sites. Growth analysis of the *pwp2-Δnsi1* mutant revealed a strong growth defect, which indicates the inability of this allele to complement the absence of Pwp2p (Supplementary Figure 4B). The mutated region in the *Δnsi1* mutant encodes the “molecular clasp” domain characteristic of β-propeller structures^29^ and may reflect a folding problem of the Pwp2-Δnsi1 mutant ﻿protein. However, the analysis of protein expression levels did not show major effects on the stability of Pwp2p (Supplementary Figure 4C).

### Pwp2p is organized in two independent and essential functional domains

**Figure 3:**
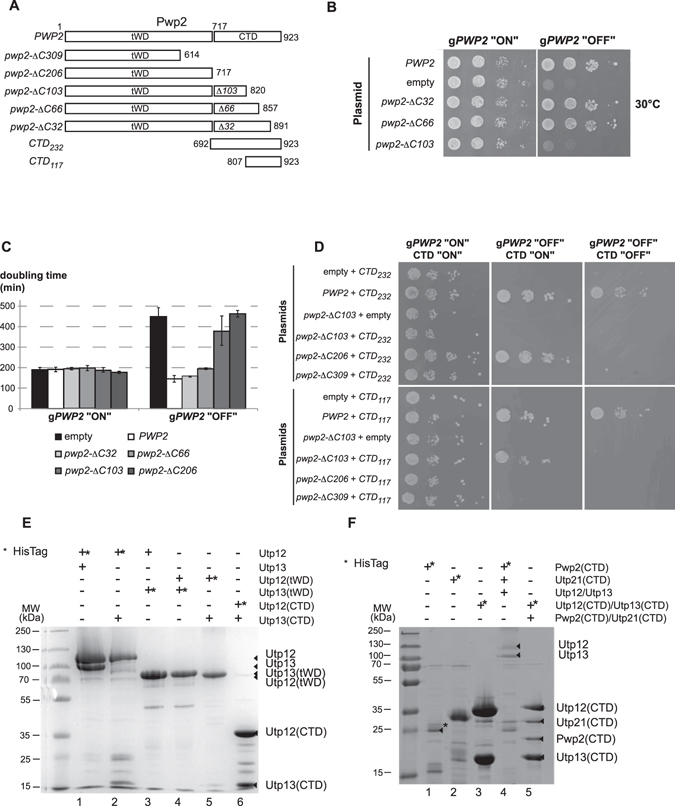
The C-terminal domain of Pwp2p is essential for growth. (**A**) Schematic representation of Pwp2p domain organization and constructs. (**B**) Drop assays of Pwp2p mutant strains. The yCMS3 strain was transformed with the backbone plasmid, the plasmid containing the wild-type *PWP2* (*PWP2*) or a plasmid containing one of the *Pwp2p* truncated forms. Serial dilutions of cells were plated either on SCG medium, allowing for the “overexpression” of a genomic HA-tagged copy of PWP2 (g*PWP2* “ON”), or in the SCD medium where the endogenous Pwp2p is “depleted” and only the Pwp2p copy encoded in the plasmid is expressed (g*PWP2* “OFF”). (**C**) Doubling time is dependent on the expression of a functional Pwp2p. Expression of the genomic copy of PWP2 (g*PWP2* “ON”) allows a similar doubling time for all strains regardless of the *PWP2* allele co-expressed. An increase in the C-terminal truncated fragment correlates with decreased cell viability (g*PWP2* “OFF”). (**D**) Complementation assay. The yCMS3 strain was co-transformed with two different plasmids, i) the empty backbone plasmid or the plasmid containing the wild-type *PWP2* or containing *Pwp2p* truncated forms (*pwp2-ΔC103*, *pwp2-ΔC206* or *pwp2-ΔC309*). The other plasmid contained the C-terminal fragments of Pwp2 complementary to the *pwp2-ΔC206* or *pwp2-ΔC103*, CTD_232_ or CTD_117_, respectively (see panel A). Expression of the CTD of Pwp2 is repressed in the presence of Doxycyclin. Serial dilutions of cells were plated in different media at 30 °C. All clones grew on SCG medium, allowing for the “overexpression” of a genomic HA-tagged copy of PWP2 (g*PWP2* “ON”) and lacking Doxycyclin (CTD “ON”). In contrast, only cells that expressed a wild-type Pwp2p or harboured the complementary coding regions of Pwp2p were able to grow in glucose-containing medium (g*PWP2* “OFF”) in the absence of Doxycyclin (CTD “ON”). Finally, the presence of Doxycyclin represses the expression of the CTD of Pwp2p (CTD “OFF”) and only cells containing a wild-type Pwp2p grow in SCD medium (g*PWP2* “OFF”). (**E**) Utp12p-Utp13p interaction is mediated by their respective C-terminal domains. *Indicates the His-tagged protein. (**F**) The C-terminal domain of Pwp2p and Utp21p are sufficient for the association with the Utp12p:Utp13p dimer. ^$^Corresponds to a regular contaminant from *E. coli*.

Our results together with published data^31^ indicate that Pwp2p has two structurally identified domains. We next explored the functional role of both domains *in vivo*. To this end, a series of C-terminal truncation mutants of Pwp2p were generated at positions encoding residue R614 (*−ΔC309*), L717 (*−ΔC206*), A820 (−*ΔC103*), E857 (*−ΔC66*), and E891 (*−ΔC32*) (Fig. 3A). The yeast strain YCMS3-1a containing the endogenous *PWP2* allele expressed under a galactose promoter was transformed with the plasmids containing different truncated versions of *PWP2*. The individual expression of each mutant protein was checked by WB analysis and it did not show any difference in their expression level or stability with the exception of *pwp2-ΔC103* (Supplementary Figure 4C). The expression of *pwp2-ΔC32* alone did not cause any effects on growth at any tested temperature (Fig. 3B,C, and Supplementary Figure 5A), suggesting that deletion of the last 32 aa had no major consequence with respect to protein function. Further removal of the last 66 aa of Pwp2p (*pwp2-ΔC66*) caused a growth defect at 30 °C, which increased slightly at lower temperatures (Fig. 3B,C, Supplementary Figure 5A and B), and may reflect a partial loss of protein function. The expression of the Pwp2p mutants *pwp2-ΔC103*, *−ΔC206* (tWD domain), and −*ΔC309* resulted in non-functional proteins that were unable to support cell growth in the absence of a wild-type copy of the *PWP2* gene (Fig. 3B, Supplementary Figure 5A). This result indicates that the expression of the tWD domain of Pwp2p (*pwp2-ΔC206)* is not sufficient for cell viability.

Next, in order to test whether the Pwp2p-tWD and C-terminal domains have independent functions besides independent folding, we constructed plasmids encoding the C-terminal domain of Pwp2p complementary to *pwp2p-ΔC103* (CTD_117_) and to *pwp2-ΔC206* (CTD_232_) (Fig. 3A and Supplementary Figure 3). These two polypeptides were expressed under the control of a Tet repressible promoter. The Pwp2-conditional yeast strain (YCMS3-1a) was transformed with a plasmid containing either CTD_232_ or CTD_117_ in combination with a plasmid containing the wild-type PWP2, the *pwp2* truncated mutants (ΔC103, ΔC206, ΔC309), or the backbone plasmid (empty). In all cases, the expression of the wild-type Pwp2p was enough to allow cell growth (Fig. 3D and Supplementary Figure 5C). In contrast, the expression of truncated Pwp2p proteins and the respective complementary CTD fragment was required to restore cell growth under depletion of the endogenous copy of Pwp2p (Fig. 3D and Supplementary Figure 5C). Expression of the corresponding CTD fragments is essential, because repression of the Tet promoter by the addition of Doxycycline did not allow cell growth (Fig. 3D and Supplementary Figure 5C). Altogether, these data indicate that the C-terminal domain of Pwp2p has an essential role *in vivo*.

As Pwp2p bridges Utp12p-Utp13p association to Utp21p-Utp6p-Utp18p, we next explored which domain of Pwp2p is necessary in the network of interactions. Along this line, we also defined the core interacting region of Utp12p and Utp13p. To this end, several constructs were designed and tested in an *in vitro* co-expression and pull-down assay (Supplementary Figure 6A). The N-terminal and C-terminal domains of Utp12p and Utp13p harbouring respectively the tandem WD-repeat domains, and the interaction domains were cloned separately. Indeed, a clear signature for a tandem WD-repeat region can be identified at the N-terminus while an α-helical region spans the C-terminal end. This was recently confirmed with the crystal structure of the ctUtp21 CTD in interaction with ctPwp2 C-terminus^31^. Interacting proteins were co-expressed and purified using the His-tag placed at the N-terminus of one of them. We first confirmed that full-length Utp12p and Utp13p interact efficiently (Fig. 3E lane 1). The presence of the C-terminal domains of Utp12p and Utp13p but not their tandem WD domains is required for the interaction (Fig. 3E compare lanes 1 and 2 with 3 and 4). Moreover, the interaction between Utp12p and Utp13p can be restricted to their respective C-terminal domains (Fig. 3E lane 6 and Supplementary Figure 7). This finding is in agreement with recently published data on *Chaetomium thermophilum* Utp12 and Utp13^31^.

Next, to map the minimal region necessary and sufficient for the association of the heterodimer Utp12p:Utp13p with Utp21p and Pwp2p, we performed pull-down assays using a combination of constructs including full-length Utp12p and Utp13p with the CTD region of Pwp2p and Utp21p (Fig. 3F). We first expressed the individual CTD of Pwp2p and Utp21p and we coexpressed the Utp12p and Utp13p CTDs as controls (Fig. 3F, lane 1–3). The coexpression of Pwp2p and Utp21p CTDs did not give rise to soluble complex (data not shown). We next coexpressed full-length Utp12p:Utp13p complex in the presence of Pwp2p and Utp21p CTDs (Fig. 3F lane 4). The four subunits could be identified on a SDS-PAGE by Coomassie Blue staining after a pull-down. The identification of the bands was confirmed by mass spectrometry analysis. Finally, a minimal core-interacting ensemble was found by coexpression of the Pwp2p, Utp21p, Utp12p and Utp13p CTDs (Fig. 3F lane 5).

These results indicate that the C-terminal domains of Pwp2p and Utp21p are engaged in a quaternary interaction with the proteins Utp12p and Utp13p, most possibly established through the interaction of their CTDs, in agreement with recently published data on the orthologous species *Chaetomium thermophilum* ^31^.

### A full-length Pwp2p is required for proper Utp21p localization and 18S rRNA maturation

**Figure 4:**
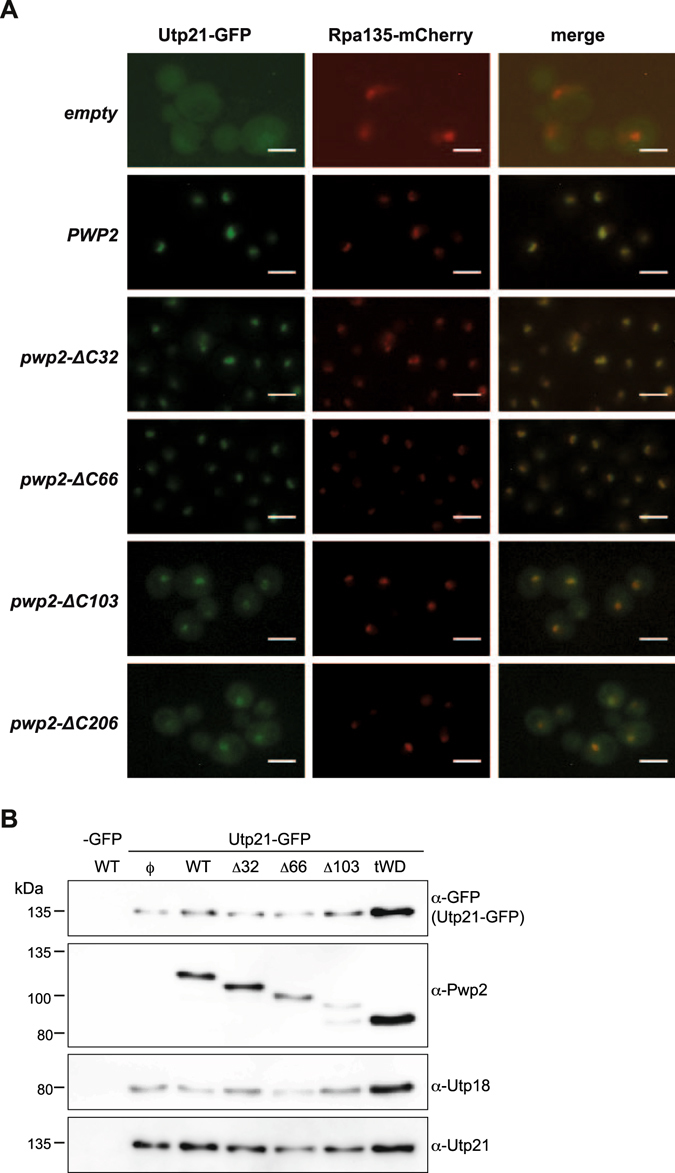
Localization of Utp21p and stability of UTP-B components under expression of different *PWP2* alleles. (**A**) Subcellular localization of Utp21p-GFP under expression of different *PWP2* alleles. A functional Pwp2p is required for the nucleolar localization of Utp21p. The strain yCMS5-1a (containing the alleles *GAL::HA-PWP2*, *UTP21-GFP*, and *RPA135*-mCHERRY at the endogenous *loci*) was transformed with plasmids encoding the Pwp2p wild-type protein or the truncated forms of Pwp2p (*pwp2-ΔC32*, *pwp2-ΔC66, pwp2-ΔC103, or pwp2-ΔC206*). Exponentially growing cells were shifted from galactose to glucose-containing medium and further cultured for 12 h in exponential growth conditions. In both cases, cells were harvested and processed for fluorescent microscopy analysis as indicated in the Methods. From left to right: Utp21p:GFP (green), Rpa135p-mCherry (red), and merged images. Scale bars represent 4 µm. (**B**) The yeast strains yMJH1-1a harbouring the *PWP2* wild type and yCMS4-1a (Utp21p-GFP) plasmids containing the Pwp2p wild type or one of the Pwp2p mutants (*pwp2-ΔC32*, *pwp2-ΔC66*, or *pwp2-ΔC103)* were cultivated in SCD-U medium for 14 h. Immuno-affinity purification was performed as described in Methods and 25% from the IPs were analysed by western blotting and revealed with antibodies against GFP, Pwp2p, Utp18p, and Utp21p.

Because the tWD domain of Pwp2 *in vitro* is not sufficient for the association of Utp12:Utp13 heterodimer with the core complex of UTP-B, we have then investigated the effect of C-terminal residues of Pwp2p on Utp21p localization. We created Pwp2p-conditional strains containing an Utp21p GFP tagged (YMCS4-1a) and an additional mCherry tag in the second largest subunit of the RNA polymerase I (Rpa135p) as nucleolar marker (YCMS5-1a). These strains were transformed with plasmids containing different alleles of *PWP2* (*PWP2, pwp2-ΔC32, pwp2-ΔC66, pwp2-ΔC103, and pwp2-ΔC206*). Cells were cultured in glucose containing medium for 12 h to deplete the endogenous *PWP2* and then analysed by fluorescence microscopy. The localization of Utp21p was not affected when the WT Pwp2p or the C-terminal truncated versions *pwp2-ΔC32* or *pwp2-ΔC66* were expressed (Fig. 4A and S8). In contrast, expression of *pwp2-ΔC103* and *pwp2-ΔC206* prevents Utp21p nucleolar localization and the GFP signal is diffusely observed in the nucleus and cytoplasm. We tested the stability of Utp21p-GFP and its association with Pwp2p and Utp18p by affinity purification of the Utp21p-GFP tagged protein (see Methods). In any case, the stability of Utp21p-GFP was not compromised and Utp21p remained associated with Utp18p (Fig. 4B). Surprisingly, although the Utp21p is associated with Pwp2p-ΔC206 mutant (Fig. 4B), its nucleolar localization was impaired (Fig. 4A). These results would suggest that formation of the core complex of UTP-B is not enough for the nucleolar localization of Utp21p and may require the entire UTP-B complex formation.

**Figure 5:**
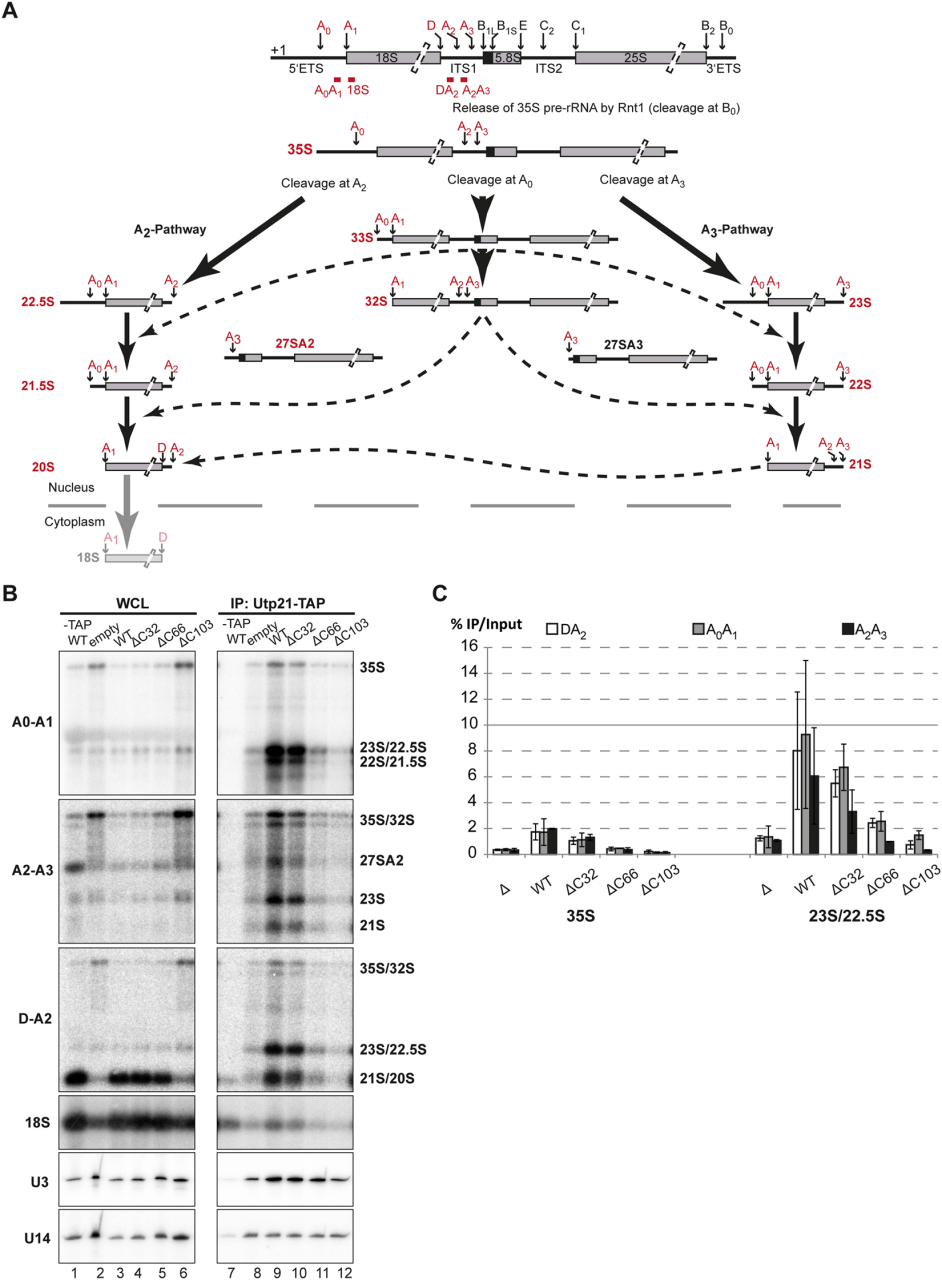
Association of Utp21p with pre-rRNAs under the expression of different *PWP2* alleles. (**A**) Schematic representation of the rRNA processing pathway during maturation of the 40S ribosomal subunit in yeast. Probes used in northern blot analysis are depicted in red. The rRNAs that were detected in our assays are labelled in red. Alternative options for an initial cleavage of the 35S at positions A0, A1, A2, or A3 is shown. All possible cleavage options during the maturation of the 40S particle are depicted in the A2 and A3 pathways. (**B**) The yeast strain yCMS3-1a harbouring empty plasmid, wild-type *PWP2* or truncated forms of the gene *PWP2*, were cultivated in SCD-U medium. Twelve percent from the WCL and 16.5% from the Utp21p-TAP-purified fraction were analysed (with the exception of the yMJH1-1a, where 25% of the WCL was analysed). The different rRNA species were resolved on either agarose gels (top three panels) or urea-acrylamide gels (two panels at the bottom) and analysed by northern blotting using the oligonucleotides: #2921 (upper panel), #207 (second panel from the top), #3839 (third panel from the top), #3468 (four panel from the top) or #3470 (lower panel). The hybridisation sites of the different probes are predicted in panel A. The apparent size of the detected rRNAs is indicated at the right side. (**C**) RNAs co-purified with Utp21p-TAP were compared with the total RNAs present in cell lysates.

Because the absence of nucleolar localization of Utp21p in some Pwp2p mutants could indicate a defect in the association of Utp21p with the pre-rRNA, we next investigated the impact of Pwp2p CTD deletion on the association of Utp21p with pre-rRNAs. To this end, yeast cells containing the Utp21p-TAP-tagged protein and harbouring the mutant genes *pwp2-ΔC32*, *pwp2-ΔC66*, *pwp2-ΔC103*, wild-type *PWP2*, or the plasmid backbone alone, were cultured for 14 h in glucose-containing medium to allow for the depletion of the genomic-encoded copy of Pwp2p (see Methods). Affinity purification against Utp21p-TAP was performed (see Methods) on the cell extracts and a yeast strain lacking the TAP-tagged Utp21p but containing a wild-type version of *PWP2* grown under the same conditions was used as a control for the purification. Total RNAs from the whole cell lysate (WCL) or from the Utp21p co-purified RNAs were analysed by northern blot using different probes at relevant sites of the 18S processing pathway (Fig. 5A). RNAs present in the WCL were enriched in 35S precursor upon Pwp2p depletion and upon expression of *pwp2p-ΔC103* (Fig. 5B, lane 2 and 6). Accordingly, in both strains, a large depletion of the 20S pre-rRNA was also observed. On the other hand, RNA analysis of *pwp2-ΔC32* and *pwp2-ΔC66* showed a similar pattern for the 20S precursors to that of the wild-type strain (Fig. 5B, compare lanes 4 and 5 with 1 and 3). In all cases, the expression level of the U3 and U14 snoRNAs remained unchanged. Expression of wild-type Pwp2p or *pwp2-ΔC32* maintained the association of Utp21p with all possible 20S pre-rRNA precursors (Fig. 5B, compare lanes 9 and 10 with 8, 11 and 12). Remarkably, under wild-type Pwp2p expression, the recovery of pre-rRNA species between the 23S and 21S with Utp21p-TAP was three times higher than for 35S pre-rRNA (Fig. 5C). This may indicate an increased affinity of UTP-B for the pre-rRNA intermediates and its commitment during the maturation steps of 20S pre-rRNA precursors. In contrast, 20S pre-rRNA purification efficiency decreased at least six times when compared with 23S pre-rRNAs and could indicate the release of UTP-B after pre-rRNA cleavage at the A1 and A2 sites (Fig. 5C). ﻿These data suggest that UTP-B has a role all along the nuclear assembly pathway of pre-40S particles.

Regarding the other mutants, the association of Utp21p with 20S pre-rRNA precursors was lower in Pwp2p-depleted cells or under expression of the *pwp2-ΔC103* and *pwp2-ΔC66* mutants in comparison with the wild-type situation (Fig. 5B, lanes 9, 10 and 11 and Fig. 5C). Unexpectedly, the truncation of the last 103 aa or the last 66 aa of Pwp2p (*pwp2-ΔC103* and *pwp2-ΔC66* mutants) showed a similar decrease in the association of Utp21p with the 20S pre-rRNA precursors, which was not reflected in their different growth phenotypes. This result suggests a reduced affinity of Utp21p for pre-rRNAs when either the last 66 or 103 aa of Pwp2p are missing. Nevertheless, the levels of U3 snoRNA associated with Utp21p were reduced after depletion of Pwp2p or in the presence of Pwp2p-ΔC103 (Fig. 5B bottom panels) but not when Pwp2p-ΔC66 was expressed. Altogether, the expression of the tWD domain of Pwp2p (*e.g. pwp2-ΔC206*, *pwp2-ΔC66*) is sufficient for its association with Utp21p *in vivo* but not to maintain binding of Utp21p to pre-rRNAs and/or tether Utp21p in the nucleolus. Moreover, our data show an overall enrichment of Utp21p with late nuclear pre-18S rRNA species, suggesting a role of the UTP-B complex all along the assembly pathway of pre-40S particles inside the nucleus.

### Targeting of UTP-B by SSU-processome subunits requires the Pwp2p-C terminal domain

**Figure 6:**
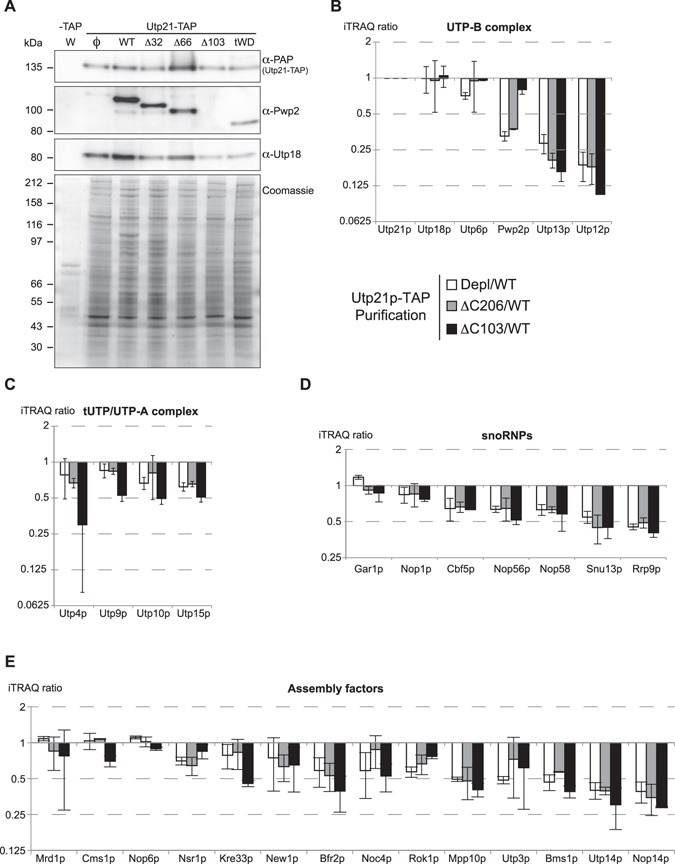
iTRAQ analysis of Utp21p-TAP affinity-purified particles under expression of different alleles of PWP2. The yeast strains yMJH1-1a harbouring the *PWP2* wild type and yCMS3-1a harbouring the *PWP2* wild type or the Pwp2p mutants (*pwp2-ΔC32*, *pwp2-ΔC66*, *pwp2-ΔC103*, or *pwp2-ΔC206*) were cultivated in SCD-U medium for 14 h. (**A**) Ten percent of the IP products were analysed by western blotting and revealed with the indicated antibodies. Twenty-five percent of the IP products were analysed by Coomassie staining. (**B**–**E**) Proteins in the Utp21p-TAP-purified samples were identified and quantitated by MS. Isobaric labelling of peptides (iTRAQ, see Methods) was used to compare the levels of the individual proteins in the Utp21p-TAP purification from cells containing a mutant Pwp2p with the respective levels in the Utp21p-TAP fractions from wild-type cells. The bar graphs represent the ratios for the indicated purifications represented on a logarithmic scale. The average of ratios for proteins identified in two independent experiments is shown for proteins belonging to the UTP-B complex (**B**), the tUTP/UTP-A complex (**C**), snoRNPs (**D**) and general SSU-processome factors (**E**).

The expression of the tWD domain of Pwp2p prevents the nucleolar localization of Utp21p and its association with the pre-rRNAs. Aiming to define the impact of the tWD domain of Pwp2 in the assembly of UTP-B and the association of UTP-B with AFs, we characterised the Utp21p-associated proteome in presence of truncated Pwp2p mutants. As previously described, cells containing the Utp21p-TAP-tagged protein and harbouring the corresponding mutated *PWP2* genes (*pwp2-ΔC32, pwp2-ΔC66, pwp2-ΔC103, and pwp2-ΔC206)* or a wild-type *PWP2* were cultivated for 14 h under depletion conditions for endogenous Pwp2p. Immuno-purified proteins were analysed by WB and q-MS. WB analysis revealed a band corresponding to Utp21p-TAP indicative of its stability under the expression of the different PWP2 alleles (Fig. 6A). As previously shown, most of the Pwp2p truncation mutants were co-purified with Utp21p as detected by WB. However, the amount of *pwp2-ΔC103* mutant in the Utp21p-TAP pull-down was largely decreased in comparison with that of other Pwp2p mutants. Nevertheless, the association of Utp21p with Utp18p was not affected under any circumstance as expected by its Pwp2p-independent association.

Consistent with the expression level and stability of Pwp2p-ΔC103 (aa 1–820), q-MS analysis showed a reduced association between Pwp2p and Utp21p when compared with Pwp2p-ΔC206 (aa 1–717). The length of the Pwp2p C-terminal truncation correlated with a reduction in the association of Utp21p with Utp12p and Utp13p, while the association of Utp6p and Utp18p with Utp21p (Fig. 6B) was not affected upon expression of any Pwp2p mutant. Altogether, these results confirm a Pwp2-independent assembly of the heterotrimeric complex Utp21p-Utp6p-Utp18p and an important role of the CTD of Pwp2p in the association of Utp12p and Utp13p with the core complex of UTP-B *in vivo*. In agreement with *in vitro* data, our data indicate that Pwp2p-(717–923) is involved in a network of interactions with Utp21p, Utp12p, and Utp13p.

Finally, we investigated the influence of C-terminal truncations of Pwp2p on the ability of UTP-B to associate with other SSU-processome components. The truncation mutants mildly affected the association of Utp21p with t-UTP components (Fig. 6C) and snRNP protein components (Fig. 6D). Further truncation of Pwp2p-CTD correlated with an increased reduction in the association of Utp21p with several AFs (Kre33p, Bfr2p, Mpp10p, Bms1p, Utp14p and Nop14p). The association of Utp21p with a small set of proteins including Nop6p and Mrd1p was not affected when the Pwp2p C- terminal truncated mutants were expressed (Fig. 6E).

In conclusion, the data indicate an important role of Pwp2p-CTD in the assembly of UTP-B which may be necessary for the association of Utp21p with some AFs of the SSU-processome. Nevertheless, Utp21p associates in a Pwp2p-independent manner with a small subset of AFs.

## Discussion

Pwp2p is a protein subunit of UTP-B and essential for the stable assembly of a large set of proteins forming the SSU-processome^5, 6, 8^. In this article, we explore the structure-function relationship of Pwp2p domains in UTP-B and SSU-processome assembly. The N-terminal tandem WD-repeat domain of Pwp2p folds as a tandem β-propeller structure, similar to the one found at the N-terminus of Utp21p, another UTP-B complex subunit^27^. Both, the tWD domain and the C-terminal domain of Pwp2p are required for the function of Pwp2p, but they have independent roles. In agreement with *in vitro* published results^16^, Pwp2p has no influence on the formation of the Utp21p:Utp6p:Utp18p heterotrimer. The tWD domain of Pwp2p stabilizes the association with Utp21p and it is necessary to form the core complex of UTP-B. However, this sole region is not sufficient for the stable incorporation of the Utp12p:Utp13p dimer. Indeed, our data also indicate that the CTD of Pwp2p promotes the association of the whole UTP-B complex through an interaction network between the C-terminal regions of Utp12p, Utp13p, Utp21p, and Pwp2p. This proposal is supported by the recently published crystal structure of the heterotetramer composed of Utp12, Utp13, Utp21, and Pwp2 C-terminal domains from *C. thermophilum* ^31^. Thus, this organization is conserved across species.

The cellular localization of Utp21p suggests a cytosolic formation of the core-complex that cannot be efficiently imported into the nucleus in the absence of Pwp2p. Moreover, the presence of the tWD domain of Pwp2p is not sufficient to trigger nuclear import of the core-complex. Then, either the association of the dimer Utp12p:Utp13p or the CTD of Pwp2p itself is a key event for the nucleolar localization of the UTP-B components. Even though the last 66 residues of Pwp2p are not necessary for the *in vitro* interaction of the C-terminal domains of Utp12, Utp13, Utp21 and Pwp2^31^, their deletion *in vivo* causes a slow growth phenotype, and disturbs the incorporation of the Utp12p:Utp13p dimer and SSU-processome formation. It is likely that this region of Pwp2p further stabilizes the association of the dimer Utp12p:Utp13p, in *S. cerevisiae* at least. Altogether, the data suggest that Pwp2p has a central position in UTP-B architecture.

As suggested by others^12^, the incorporation of UTP-B in the SSU-processome would occur through initial contacts of Utp6p and Utp18p with the pre-rRNA. Because our data suggest that UTP-B formation is a key event for its stable association with pre-rRNA and for the SSU-processome formation, most likely the association of Utp6p and Utp18p with the pre-rRNA would take place after assembly of the UTP-B complex. The RNA chaperone role attributed to UTP-B^13^ may involve multiple contacts with the pre-rRNA that can be established during maturation of the SSU processome. In support of this hypothesis, the UTP-B components Utp18p and Utp13p are associated with the 18S rRNA and ITS1 regions, respectively^12^, and we observe the association of Utp21p with all possible precursors of the 20S pre-rRNA.

Finally, the co-expression of the two structural domains of Pwp2p complements the function of wild-type Pwp2p. It is likely that the CTD of Pwp2p is dedicated to stabilize the dimer Utp12p:Utp13p within the UTP-B complex even in the absence of the tWD domain. Thus, we suggest that the incorporation of the dimer Utp12p:Utp13p is not sufficient to trigger SSU-processome formation and would require the presence of the tWD domain of Pwp2p. Furthermore, a large set of factors, AFs participating in different steps of pre-20S maturation^12, 32^, including the Mpp10 complex, the Bms1p-Rcl1p dimer, and the U3 snoRNP among others cannot associate with the pre-rRNA in the absence of Pwp2p^6–9, 22^. It is likely that direct protein-protein interactions between AFs and specific UTP-B components are responsible for the hierarchical recruitment of AFs to the SSU-processome but the precise interactions are so far elusive. Since we cannot hypothesize that each UTP-B subunit has an exclusive function/role, the tWD domain of Pwp2p might be required for both the interaction with Utp21p and the recruitment of other AFs. In this regard, the AFs Mrd1p and Nop6p might directly interact with Utp21p, because their association does not depend on SSU-processome formation. However, this observation is in contrast with the Pwp2p-independent association of Mrd1p with the pre-rRNA^32^. We propose that UTP-B constitutes a platform/node for the association of some SSU components and probably with pre-RNA segments.

## Methods

### Yeast strains and microbiological procedures

Oligonucleotides, plasmids and yeast strains used in this work are listed in Tables S1, S2, and S3. The conditional mutant strain for *PWP2* expression under the control of a GAL1 promoter was generated by a one-step PCR strategy using the oligonucleotides o82-o84 to amplify the KANMX::GAL::HA cassette from pFA-KANMX6-PGAL1^33^. The PCR product was used to transform the yeast strain BY4741 (Euroscarf) to obtain the strain YMHJ-1a. The gene locus *YLR409c* fused to the TAP coding sequence was amplified from the strain BY4741 *YLR409c-TAP*-(EUROSCARF) with the oligonucleotides o32-o33 and the PCR product was transformed into the strain YMHJ1-1a to obtain the strain YCMS3-1a. Strain YCMS4-1a containing the GFP-tagged Utp21p was obtained by the one-step PCR strategy using the oligonucleotides o1-o2 to amplify the plasmid pYM44^34^, and the PCR product was used to transform the strain YMJH-1a. Strain YCMS5-1a contains the mCherry tag fused to the second largest subunit of Pol I (Rpa135p). This strain was obtained by a one-step PCR strategy using the oligonucleotides #1203–#1204 to amplify the plasmid pRJD1, and the PCR product was used to transform the strain YCMS4-1a.

Yeast cells were cultured in YPG (1% yeast extract, 2% bacto peptone, and 2% galactose) or YPD (1% yeast extract, 2% bacto peptone, and 2% glucose). In order to avoid loss of plasmids harbouring the *pwp2* mutants, yeast cells were cultivated in minimal media (YNB and a drop out of the required supplements according to the strain auxotrophy) containing either 2% galactose (SCG) or 2% glucose (SCD). The growth on galactose- or glucose-containing media allowed the overexpression or repression, respectively, of the genomic-encoded copy of *PWP2 (gPWP2 “ON” and “OFF”)*. Cell cultures were grown for the indicated times.

### DNA cloning

The *PWP2* gene from *S. cerevisiae* was amplified by PCR from genomic DNA and cloned into the *BamH*I and *Nde*I restriction sites of a modified pET-15b (Novagen) plasmid^35^, allowing for expression of a fusion protein containing a hexa-histidine tag and TEV cleavage site at the N-terminus of Pwp2p. Point mutations were introduced by PCR. *PWP2* containing plasmids for *in vivo* experiments contained a DNA fragment including 486 bp upstream the ATG and 314 bp downstream from the STOP codon for the normal expression of Pwp2p. The different mutant alleles were obtained as indicated in Table S2. All constructs were sequenced to ensure the absence of non-desired mutations.

### Expression and purification of Pwp2p (1–716)

Overexpression of the recombinant yeast Pwp2p (residues 1–716) was carried out in *Escherichia coli* BL21 (DE3) Rosetta cells. Cultures were grown in Terrific Broth at 37 °C supplemented with the relevant antibiotics. Induction of protein expression was initiated by adding IPTG at a final concentration of 1 mM when cell density reached an OD600 of 1.5–2, and expression continued for 15 h at 15 °C. Cells were harvested by centrifugation at 4,000 *g* for 15 min, and the cell pellet was frozen at −20 °C overnight. Bacteria were then lysed by sonication and centrifuged at 50,000 *g* for 45 min at 4 °C. The supernatant was applied to a cobalt-affinity gel for 1 h at 4 °C. After extensive washes with a wash buffer containing 25 mM Tris, pH 8.0 and 300 mM NaCl, the protein was eluted with a gradient of imidazole (0–500 mM). Peak fractions corresponding to the protein were then pooled and diluted in 25 mM Tris, pH 8.0 and 150 mM NaCl. A second chromatography step was performed using a heparin affinity column (GE, HiTrap Heparin-HP). The protein eluted around 250 mM NaCl. Peak fractions were pooled, concentrated and applied to a size exclusion chromatography HiLoad 16/60 S75 prepgrade column (GE) and eluted with 25 mM Tris, pH 8.0 and 150 mM NaCl.

### Crystallization and structure determination

Prior to crystallization, the protein was concentrated to 20 mg/ml in a buffer containing 25 mM Tris-HCl, pH 8.0 and 50 mM NaCl. The protein was crystallized at 20 °C by the sitting drop method against a reservoir containing 5% polyethylene glycol 6000 and 0.1 M magnesium sulphate. The crystals were cryo-protected in the crystallization solution supplemented with 10% glycerol, flash-frozen in liquid nitrogen and maintained at 100K in a nitrogen cryo-stream during data collection.

Crystals belonged to the space group *P*2_1_2_1_2_1_ with unit cell dimensions a = 70.8 Å, b = 76.04 Å and c = 191.35 Å, containing one molecule per asymmetric unit and diffracted up to 2.54 Å (Table 1). Datasets were reduced using XDS^36^. The structure of Pwp2p (1–716) was solved by molecular replacement using the BALBES server^24^. A good solution was found using PDB 2PBI (chain B, residues 55 to 351). The initial model was built using Bucanneer^37^. Iterative cycles of manual building using COOT^38^ and refinement with BUSTER 2.10^26^ were performed. The final model was refined to Rfree/Rfactor values of 23.9%/19.2% and had good stereochemistry. It has been deposited in the PDB under the code 5I2T (Table 1).

### Co-expression experiments

Pairwise co-expression was performed by transformation of Rosetta2 cells with modified pET-15b in which *UTP12* or *UTP13* constructs were cloned; pCDF-Utp13p and pCDF-Utp12p constructs were also used for co-expression. Cells were plated on Chloramphenicol (33 μg/ml), Ampicillin (100 μg/ml) and Streptomycin (50 μg/ml). Cells grown at 37 °C in Terrific Broth were induced by addition of 1 mM (final concentration) IPTG and incubated at 15 °C overnight. Harvested cells were re-suspended in 1.5x PBS, 1 mM magnesium acetate, 0.1% NP-40, 20 mM imidazole and 10% glycerol and sonicated. A fraction of the crude extract was saved at this stage and boiled in Laemmli buffer; the remaining extract was centrifuged, and the supernatant was incubated with cobalt-affinity resin for 30 min at 4 °C. Beads were washed three times in the same buffer. Approximately 5% of the crude extract and 15% of the bound fraction was analysed on a 15% SDS-PAGE and revealed by Coomassie Blue staining.

For co-expression of Pwp2p, Utp21p, Utp12p, and Utp13p, a bi-cistronic construct containing *UTP12* and *UTP13* was designed and cloned into a modified pET-28b plasmid. *PWP2* and *UTP21* constructs were cloned into modified pET-15b and pCDF vectors for co-expression. The three plasmids were co-transformed and protein expression was performed as described above.

### Fluorescence microscopy experiments

The strains YCMS4-1a (containing the *UTP21*::GFP) and YCMS5-1a (containing *UTP21*::GFP and *RPA135*::mCHERRY) were transformed with plasmids containing the wild type *PWP2*, or one of the the PWP2 alleles, *pwp2-ΔC32*, *pwp2-ΔC66, pwp2-ΔC103, or pwp2-ΔC209 (tWD domain)*. Cells were cultured in synthetic medium (SC) containing galactose, shifted to SC containing glucose and cultured for a further 12 h. Exponentially growing cells from YCMS4-1a-derived strains were fixed with 0.1 volumes 37% formaldehyde for 1 h under growth conditions. Cells were collected by centrifugation at 3,000 rpm for 3 min at RT, the supernatant was discarded and the cell pellet was washed in 1 ml 0.1 M potassium phosphate, pH 7.5. To obtain spheroplasts, the cell pellet was resuspended in 1 ml 0.1 M potassium phosphate, pH 7.5 containing 50 µg/ml Zymolyase 100 T and 2 µl/ml β-mercaptoethanol. The cell suspension was incubated for 45 min at 30 °C with gentle shaking at 180 rpm. The spheroplasts were collected by centrifugation at 2,000 rpm for 3 min at RT and resuspended in 1 ml 1x TBS (136 mM NaCl; 2.7 mM KCl; 12.5 mM Tris, pH 7.4). Cells (50 µl) were spotted on diagnostic microscopy slides previously coated with poly-L-lysine (Sigma) and incubated for at least 5 min to allow cell adhesion. Excess cells were removed and washed three times with 50 µl 1x TBS. DNA was counterstained with 3 µl 0.1 µg/µl DAPI in Mowiol [13% (w/v) Mowiol (Calbiochem); 33% glycerol (w/v); 66 mM Tris, pH 8.5). Prepared slides were stored at 4 °C in the dark. In parallel, exponentially growing cells from YCMS5-1a-derived strains were collected by centrifugation and dispersed on slides containing SC-D and 2% agarose for *in vivo* microscopy analysis. Images were captured during 10 min in an inverted Axiovert 200 microscope (Zeiss) using an AxioCam MRm and the AxioVision software. Image processing was carried out with ImageJ software. Exposure times were the same for all yeast mutants (17 s GFP, 4 s mCherry, 40 ms DIC).

### Analysis of generation times

Strains containing the empty YCplac33 plasmid or a plasmid containing a PWP2 allele were precultivated in selection medium (lacking uracil) and containing 2% galactose. The generation time was determined by measuring the increase in cell density upon cultivation in 96-well plates at 30 °C or 37 °C in selection medium containing either galactose (gPWP2 “ON”) or glucose (gPWP2 “OFF”) using a Tecan (Infinite 500) reader. Alternatively, the generation time at 23 °C was determined by measuring the increase in cell density upon cultivation in 20-ml cultures in selection medium containing either galactose (gPWP2 “ON”) or glucose (gPWP2 “OFF”).

### TCA-urea extraction

Cellular extracts using the TCA-urea protocol were prepared from five optical units of exponentially growing cells. Cells were collected at 3,000 rpm for 3 min at room temperature and resuspended in 1 ml of cold water and 150 µl of DB1 (7.5% v/v β-Mercaptoethanol and 1.85 M NaOH) were added. Samples were incubated for 15 min on ice, 150 µl of DB2 (55% TCA) were added and the samples were incubated for a further 10 min on ice. Afterwards, cells were centrifuged at 14,000 rpm for 10 min at 4 °C and the supernatant was discarded. Pellets were resuspended in 150 µl HU buffer (5% w/v SDS; 200 mM Tris, pH 6.8; 1 mM EDTA; 1.5% v/v β-mercaptoethanol; 48% w/v urea and 0.1% w/v bromophenol) and the pH was neutralized with ammonia. Samples were boiled at 85 °C for 20 min and finally, centrifuged at 14,000 rpm for 1 min at room temperature.

### Affinity purification of Utp21p-TAP using IgG-coupled magnetic beads and semi-quantitative mass spectrometry analysis of Utp21p-associated proteins

Yeast cells cultured as previously indicated were harvested, and affinity purifications of Utp21p-TAP and associated protein-containing particles were carried out as described in ref. 39 using buffer PM (150 mM KOAc; 20 mM Tris-HCl, pH 8.0; 5 mM MgCl_2_; 1 mM DTT; 0.2% w/v Triton) instead of MB. Eluted fractions of the affinity purifications were further processed for semi-quantitative mass spectrometric analysis as previously described^40^ and using equal amounts of total protein. Ten percent of the eluted material was collected to perform western blot (WB) analysis. Data were normalized by setting the respective Utp21p-TAP iTRAQ ratios to one.

### Affinity purification of RNAs associated with Utp21p-TAP using IgG-coupled sepharose beads

Yeast cells cultured in glucose containing medium as previously indicated were harvested after 14 h from exponentially growing cultures. The affinity purifications of Utp21p-TAP and associated proteins were performed as described in ref. 23 with slight variations and using equal amounts of total proteins. We used buffer PM (150 mM KAc; 20 mM Tris-HCl, pH 8.0; 5 mM MgCl_2_; 1 mM DTT; 0.2% w/v Triton) instead of A200. Ten percent of the eluted sample was collected for WB analysis as a control for the immuno-affinity purification (data not shown). Ninety percent of the eluted samples were used for RNA extraction.

### Affinity purification of Utp21p-GFP using GFP-TRAP®-coupled magnetic beads

Yeast cells cultured in glucose containing medium as previously indicated were harvested after 14 h from exponentially growing cultures. The affinity purifications of Utp21p-TAP and associated proteins were carried out as described in ref. 39 with slight variations, using equal amounts of total proteins. We used the GFP-TRAP®-M from Chromotek and buffer PM (150 mM KOAc; 20 mM Tris-HCl, pH 8.0; 5 mM MgCl_2_; 1 mM DTT; 0.2% w/v Triton) instead of MB. Twenty percent of the eluted material was collected to perform WB analysis. Data were normalized by setting the respective Utp21p-TAP iTRAQ ratios to one.

### Western blot analysis

Detection of TAP tag was performed using an HRP-coupled antibody against protein A (A01435-100 Genscript). Detection of Utp21p, Pwp2p, and Utp18p was monitored using polyclonal primary antibodies generated against the untagged Pwp2p (1–716), untagged Utp21p (1–698), and untagged Utp18p by Agro-Bio, respectively. HRP-coupled anti-rabbit secondary antibody (111-035-003, Dianova) was used to detect polyclonal antibodies. Detection of Tubulin was performed with the monoclonal antibody YOL1/34 (ab6161, Abcam) and the HRP-coupled anti-rat secondary antibody (112-035-068, Dianova). Protein signals were visualized using the Chemiluminescence Western blotting reagent (Roche) in LAS-3000 (Fujiflm).

### RNA extraction and northern blotting

RNAs were extracted by hot acidic phenol/chloroform treatment as previously described^41^. Northern blotting analyses after RNA separation on formaldehyde/MOPS agarose gel (20S precursors) or urea/TBE/polyacrylamide gels (U3 and U14) were carried out as described in ref. 42. Hybridization with the radioactive labelled probes listed in Table S1 was performed as previously described^6^.

## Electronic supplementary material

Supplementary
