# Supplementary material for: Pwp2 mediates UTP-B assembly via two structurally independent domains
Source: Sci Rep. 2017 Jun 9;7:3169. doi: 10.1038/s41598-017-03034-y (PMC5466602; doi:10.1038/s41598-017-03034-y)
Supplement: Supplementary file 1 — Supplementary [file 41598_2017_3034_MOESM1_ESM.pdf]

Supplementary information

Title

Pwp2 mediates UTP-B assembly via two structurally independent domains

Fanny Boissier [1$], Christina Maria Schmidt [2$], Jan Linnemann[2], Sébastien Fribourg [1*]; Jorge Perez-Fernandez [2*]

[1] Université de Bordeaux, INSERM U1212, CNRS 5320, Bordeaux, France

[2] Universität Regensburg, Biochemie-Zentrum Regensburg (BZR), Lehrstuhl Biochemie III, D-93053 Regensburg, Germany

SUPPLEMENTAL DATA

Table S1: Oligonucleotides used in this study. Oligonucleotides used for different purposes are described. Database numbers, sequence and usage are indicated. Oligonucleotides marked with "***#***" belong to the Department of Biochemistry III at the University of Regensburg. Oligonucleotides marked with "***o***" belong to the collection of the UTP-Lab.

Col. N.	Name	5'-3'-sequence	Purpose
**#1203**	RPA135-pYM-for	CTATCCGCAATGGGTATAAGATTGCGTTATAATGTAGAGCCCAAACGTACGCTGCAGGTCGAC	Forward oligo for the mCherry tagging of Rpa135 using the plasmid pFA6a:1xmCHERRY-Hph
**#1204**	RPA135-pYM-rev	CCTTCATTTACCATTCTATATCAATTTGGAAAGAAGGGTATTTCTATCGATGAATTCGAGCTC	Reverse oligo for the mCherry tagging of Rpa135 using the plasmid pFA6a:1xmCHERRY-Hph
**#3942**	SalI_Pwp2_5	GTCGACGCGGTTCAATCTGCGGTCTGCTC	Used to eliminate the PWP2 coding region and insert a SalI site instead.
**#3943**	PstI_Pwp2_3	TGACTGCAGAAAATATTCACTGTCCTCTA	Used to eliminate the PWP2 coding region and insert a PstI site instead.
**#3949**	5'SLIC_PWP2	GTCACGACGTTGTAAAACGACGGCCAGTGAATTCGAGCTCGACTCCAAGATGCGTAAGGC	Cloning of PWP2 locus containing 5' and 3' regulatory sequences.
**#3950**	3'SLIC_PWP2	TCACACAGGAAACAGCTATGACCATGATTACGCCAAGCTTTGATAGTATGAAACGGTAGTCG	Cloning of PWP2 locus containing 5' and 3' regulatory sequences.

Col. N.	Name	5’-3’-sequence	Purpose
**#3950**	3'SLIC_PWP2	TCACACAGGAAACAGCTATGACCA TGATTACGCCAAGCTTTGATAGTAT GAAACGGTAGTCG	5'UTR_PWP23'UTR cloning
**#3951**	SalI_PWP2Fo	GTAGACAGGAGAGCAGACCGCAG ATTGAACCGCGTCGACATGAAATC CG	Cloning of *PWP2* ORF containing a SalI site for subcloning
**#3952**	PstI_Pwp2Re	TGATGTGAATGTGCATAAATAGAG GACAGTGAACTGCAGTCAAGGAA GCTC	Cloning of *PWP2* ORF containing a PstI site for subcloning
**#3953**	PstI_Pwp2_corr	TGACTGCAGTTCACTGTCCTCT	Fow primer to clone truncations
**#3162**	PWP2_XmaI_fw	TTGTTGCCCGGGATGAAATCCGAT TTCAAGTTCTCT	forward primer for sequencing of mutated pwp2 on pJPF19 and pCJPF39
**#3185**	PWP2_seq_fw2	GAGGCCATCCGATGACGA	forward primer for sequencing of mutated pwp2 on pJPF19 and pCJPF39
**#3186**	PWP2_seq_rev2	CCCATCAGTTGATACCAAAAATCT	reverse primer for sequencing of mutated pwp2 on pJPF19 and pCJPF39
**#3187**	PWP2_seq_rev3	TATCAATACGATCCTCCAAATCTGA	reverse primer for sequencing of mutated pwp2 on pJPF19 and pCJPF39
**#3999**	Pwp2_103C_SLIC	GTGCATAAATAGAGGACAGTGAAC TGCAGTCA_AGCCGTCGAGAAGAG ATATTTG	Cloning of DC103 truncation together with #3951
**#4000**	Pwp2_206C_SLIC	GTGCATAAATAGAGGACAGTGAAC TGCAGTCA_CAGATCAAAGGGATC AAATAATATC	Cloning of DC206 truncation cloning together with #3951
**#4001**	Pwp2_309C_SLIC	GTGCATAAATAGAGGACAGTGAAC TGCAGTCA_TCTTTTTAACAAGACT TCATTTGG	Cloning of DC309 truncation cloning together with #3951
**o1**	Utp21_S3	GTGAAGTTTTGCATGGGAGTGGCA GCTTTTGTGACCACCGCG_CGTAC GCTGCAGGTCGAC	TAP tagging of Utp21 using the pYM collection
**o2**	Utp21_S2	CAATCTGCCTTTGTTACTAATATAC TTGTTCTATATAATGC_ATCGATGA ATTCGAGCTCG	TAP tagging o Utp21 using the pYM collection
**o13**	PWP2_61XD_re	TTCTTCTAACAAGACTTCATTTGG	reverse primer used to introduce mutations into *PWP2*; as a result Lys at position 613 and Arg at position 614 are replaced by Asp
**o14**	PWP2_61XD_fo	CCAAATGAAGTCTTGTTAGAAGAA TTCATTGTGTCCAGAAACATGG	forward primer used to introduce mutations into *PWP2*; as a result Lys at position 613 and Arg at position 614 are replaced by Asp
**o16**	PWP1_32C	CTCGTCGTCAGATTCCATGAC	Cloning of DC32 truncation cloning together with #3953
**o17**	PWP2_66C	TTCCATGGACCCATCAGTTG	Cloning of DC66 truncation cloning together with #3953
**o26**	R140-fo2	CTTCGTCCGCCATGACGTACATGC GGGACACTTTCAAGAC	forward primer used to introduce mutations into *PWP2*; as a result Arg at position 140 is replaced by Asp

Col. N.	Name	5'-3'-sequence	Purpose
**o27**	R140-re2	GTGTCCCGCATGTACGTCATGGCG GACGAAGGGAGCAAAC	reverse primer used to introduce mutations into *PWP2*; as a result Arg at position 140 is replaced by Asp
**o32**	Utp21_2302fo	GGGGATGAAGCCTCTGTCAGAG	Movilization of C-terminal tagging coding sequences at *UTP21* locus
**o33**	Utp21_3302re	GGATTCGGAGATCCCTATCCAC	Movilization of C-terminal tagging coding sequences at *UTP21* locus
**o45**	Mut_129-30E_Fo	CAGATTTGGAAGACTCCAGATGTT AACAAACGCAGACAGTTTGCTCCC TTCGTCCG	forward primer used to introduce mutations into *PWP2* together with o47; as a result Asp at position 130 is replaced by Lys.
**o46**	Mut_130K_Fo	CAGATTTGGAAGACTCCAGATGTT AACAAACGCAGACAGTTTGCTCCC TTCGTCCG	reverse primer used to introduce mutations into *PWP2* together with o47; as a result Arg at position 129 and Lys at position 131 are replaced by Glu as well as Asp at position 130 is replaced by Lys
**o47**	Mutloop1_Re	GTTAACATCTGGAGTCTTCCAAATC TG	
**o48**	MtR505E_Fo	CGCATATGGTCCATATTTGGTGAA_ AGCCAACAAGTAGAACCTATAGAA GTTTATTCCG	forward primer used to introduce mutations into *PWP2*; as a result Arg at position 505 is replaced by Glu
**o49**	MtR505E_re	TACTTGTTGGCTTTCACCAAATATG GACCATATGCGAATTGTTTTATCCC ATGATGC	reverse primer used to introduce mutations into *PWP2*; as a result Arg at position 505 is replaced by Glu
**o50**	FLAG_pwp2A692	ATGGATTACAAGGATGACGACGAT AAGGGTACCGGATCCGCATTTGCC GCTGCTTCAAC	Forward primer used together with #3950 to amplify the coding region of last 232 aa of PWP2
**o51**	FLAG_pwp2G807	ATGGATTACAAGGATGACGACGAT AAGGGTACCGGATCC_GGTGGTTA CATAAATGAACACAAATATC	Forward primer used together with #3950 to amplify the coding region of last 117 aa of PWP2
**o82**	Pwp2_F4	GGTAGACAGGAGAGCAGACCGCA GATTGAACCGCATCCCA_GAATTC GAGCTCGTTTAAAC	forward primer for insertion of KANMX::GAL::HA cassette in the *PWP2* locus
**o84**	Pwp2_R3	GCCTGTAGACCGTACCTAAAAGGT TAGAGAACTTGAAATCGGATTTCA TGCACTGAGCAGCGTAATCTG	reverse primer for insertion of KANMX::GAL::HA cassette in the *PWP2* locus
**#205**	o2-18S	CATGGCTTAATCTTTGAGAC	Hybridize in the rDNA, 18S
**#207**	o4-A2/A3	TGTTACCTCTGGGCCC	Hybridize in the rDNA, ITS1
**#2921**	A0A1-Sonde	CCCACCTATTCCCTCTTGCTAG	Hybridize in the rDNA, 5'-ETS
**#3468**	U3-SnR17A	CCGCTAAGGATTGCGGACCAAGC	Hybridize in the U3 snoRNA
**#3570**	SnR128-U14	TCACTCAGACATCCTAGGAAGG	Hybridize in the U14 snoRNA
**#3839**	ITS1 D-A2 mid	AAGCCTAGCAAGACCGCGCA	Hybridize in the rDNA, ITS1

Table S2: Plasmids used in this study. Database numbers, name of the plasmid, contained markers for selection and the cloning procedure are indicated. Plasmids marked with "***#***" belong to the Department

of Biochemistry III at the University of Regensburg. Plasmids marked with "***p***" belong to the collection of the UTP-Lab.

#	Name	Gene	Marker	Origin	Cloning procedure
**#1**	pBS	Empty backbone	Amp	Biochemie III	
**#49**	YCplac33	empty backbone plasmid	Amp/ URA3	Biochemie III	[1]
**#1225**	pYM44	One-step PCR	Amp	Biochemie III	[2]
**#1837**	pCM182-LEU2	Empty backbone plasmid	Amp/ URA3	Biochemie III	*LEU2* marker replaces *TRP1* in pCM182 [3,4]
**p107**	pJPF19	*PWP2*	Amp	This study	*PWP2* locus including 5' and 3' regulatory regions cloned in pBluescriptKS (+/-).
**p108**	pCMS1	*pwp2-R129/K131E*	Amp	This study	PCR product obtained with oligonucleotides o45-o47 cloned in pJPF19 using the SLIC protocol
**p109**	pCMS2	*pwp2-D130K*	Amp	This study	PCR product obtained with oligonucleotides o46-o47 cloned in pJPF19 using the SLIC protocol
**p110**	pCMS3	*pwp2-E473Δnsi1*	Amp	This study	Subcloning from XX in pJPF19 using BamHI and NsiI sites.
**p111**	pCMS4	*pwp2-R505E*	Amp	This study	PCR product obtained with oligonucleotides o48-o49 cloned in pJPF19 using the SLIC protocol
**p120**	pCMS13	*pwp2-ΔC32*	Amp	This study	PCR product obtained with oligonucleotides o16-#3953 cloned in pJPF19 using blunt end ligation
**p121**	pCMS14	*pwp2-ΔC66*	Amp	This study	PCR product obtained with oligonucleotides o17-#3953 cloned in pJPF19 using blunt end ligation
**p122**	pCMS15	*pwp2-ΔC103*	Amp	This study	PCR product obtained with oligonucleotides #3999-3951 cloned in pJPF19 using the SLIC protocol
**p123**	pCJPF39	*PWP2*	Amp/ URA3	Pérez-Fernández	[SacI-HinDIII]-fragment form pJPF19 subcloned in YCplac33
**p124**	pCCMS1	*pwp2-R129/K131E*	Amp/ URA3	This study	[SalI-PstI]-fragment from pCMS1 subcloned in pCJPF39
**p125**	pCCMS2	*pwp2-D130K*	Amp/ URA3	This study	[SalI-PstI]-fragment from pCMS2 subcloned in pCJPF39
**p126**	pCCMS3	*pwp2-E473Δnsi1*	Amp/ URA3	This study	[BamHI-PstI]-fragment from pCMS3 subcloned in pCJPF39

#	Name	Gene	Marker	Origin	Cloning procedure
**p127**	pCCMS4	*pwp2-R505E*	Amp/ URA3	This study	[BamHI-PstI]-fragment from pCMS4 subcloned in pCJPF39
**p128**	pCCMS5	*pwp2-K613/R614E*	Amp/ URA3	This study	[BamHI-PstI]-fragment from pCMS5 subcloned in pCJPF39
**p136**	pCCMS13	*pwp2-ΔC32*	Amp/ URA3	This study	[BamHI-PstI]-fragment from pCMS13 subcloned in pCJPF39
**p137**	pCCMS14	*pwp2-ΔC66*	Amp/ URA3	This study	[BamHI-PstI]-fragment from pCMS14 subcloned in pCJPF39
**p138**	pCCMS15	*pwp2-ΔC103*	Amp/ URA3	This study	[BamHI-PstI]-fragment from pCMS15 subcloned in pCJPF39
**p168**	pJPF20	*pwp2-E335R*	Amp	This study	Subcloning from XX in pJPF19 using AflII and MscI sites.
**p169**	pJPF21	*pwp2-K368E*	Amp	This study	Subcloning from XX in pJPF19 using AflII and BamHI sites.
**p170**	pJPF22	*pwp2-R418E/R420E*	Amp	This study	Subcloning from XX in pJPF19 using AflII and BamHI sites.
**p171**	pJPF23	*pwp2-E473R*	Amp	This study	Subcloning from XX in pJPF19 using BamHI and NsiI sites.
**p172**	pCJPF40	*pwp2-E335R*	Amp/ URA3	This study	[BamHI-PstI]-fragment from pJPF20 subcloned in pCJPF39
**p173**	pCJPF41	*pwp2-K368E*	Amp/ URA3	This study	[BamHI-PstI]-fragment from pJPF21 subcloned in pCJPF39
**p174**	pCJPF42	*pwp2-R418E/R420E*	Amp/ URA3	This study	[BamHI-PstI]-fragment from pJPF22 subcloned in pCJPF39
**p175**	pCJPF43	*pwp2-E473R*	Amp/ URA3	This study	[BamHI-PstI]-fragment from pJPF23 subcloned in pCJPF39
**p176**	pCMS16	*pwp2-ΔC206*	Amp	This study	PCR product obtained with oligonucleotides #4000-3951 cloned in pJPF19 using the SLIC protocol
**p177**	pCMS17	*pwp2-ΔC309*	Amp	This study	PCR product obtained with oligonucleotides #4001-3951 cloned in pJPF19 using the SLIC protocol
**p178**	pCCMS16	*pwp2-ΔC206*	Amp/ URA3	This study	[BamHI-PstI]-fragment from pCMS16 subcloned in pCJPF39
**p179**	pCCMS17	*pwp2-ΔC309*	Amp/ URA3	This study	[BamHI-PstI]-fragment from pCMS17 subcloned in pCJPF39
**p180**	pCCMS24	*pwp2-G807-P923*	Amp/ URA3	This study	PCR product from o51-#3950 cloned in pCM182-LEU2-FLAG at BamHI-HindIII using the SLIC protocol
**p181**	pFA6a-kanMX6-PGAL1::3HA	One-step PCR	Amp	This Study	[5]

#	Name	Gene	Marker	Origin	Cloning procedure
**p182**	pFA6a::1xmCHERRY:Hph	One-step PCR	Amp	This Study	[2] Plasmid obtained by religation of *AleI* digested plasmid pFA6-mCHERRY-Hph (kindly provided by Brigitte Pertschy).
	pET-His-Pwp2(1-716)	One-step PCR	Amp	This Study	PCR product was cloned by LIC strategy in a modified pET-15b [6]
	pET-His-Pwp2(717-923)	One-step PCR	Amp	This Study	PCR product was cloned by LIC strategy in a modified pET-15b [6]
	pEt-Utp12p-Utp13p	One-step PCR	Kan	This Study	PCR product was cloned by LIC strategy in a modified pET-28b [6]
	pCDF-Utp21(699-939)	One-step PCR	Sm	This Study	PCR product was cloned by LIC strategy in a modified pCDF [6]
	pET-Utp21p	One-step PCR	Sm	This Study	PCR product was cloned by LIC strategy in a modified pCDF [6]
	pET-His-Utp12p	One-step PCR	Amp	This Study	PCR product was cloned by LIC strategy in a modified pET-15b [6]
	pET-Utp13p	One-step PCR	Kan	This Study	PCR product was cloned by LIC strategy in a modified pET-28b [6]
	pET-Utp13p(649-817)	One-step PCR	Kan	This Study	PCR product was cloned by LIC strategy in a modified pET-28b [6]
	pET-His-Utp13p(1-648)	One-step PCR	Amp	This Study	PCR product was cloned by LIC strategy in a modified pET-15b [6]
	pET-Utp12p(1-687)	One-step PCR	Kan	This Study	PCR product was cloned by LIC strategy in a modified pET-28b [6]
	pET-HisUtp12p(1-687)	One-step PCR	Amp	This Study	PCR product was cloned by LIC strategy in a modified pET-15b [6]
	pET-His-Utp12p(687-943)	One-step PCR	Amp	This Study	PCR product was cloned by LIC strategy in a modified pET-15b [6]
	pET-His-Utp12p(688-943)-Utp13(649-817)	One-step PCR	Amp	This Study	PCR product was cloned by LIC strategy in a modified pET-15b [6]
	pET-Kan-Pwp2p(717-943)-Utp21(699-939)	One-step PCR	Kan	This Study	PCR product was cloned by LIC strategy in a modified pET-15b [6]

Table S3: Yeast strains used in this study. Database numbers, name of the plasmid, contained markers for selection and the cloning procedure are indicated. Yeast strains marked with "***#***" belong to the Department of Biochemistry III at the University of Regensburg. Plasmids marked with "***Y***" belong to the collection of the UTP-Lab.

#	Name	Genotype	Origin
**#206**	BY4741	MATa; *his3-1*; *leu2-0*; *met15-0*; *ura3-0*	Biochemie III
**#2506**	BY4741 UTP21-TAP	MATa; *his3-1*; *leu2-0*; *met15-0*; *ura3-0*; *YLR409C*-TAP::*HIS3*MX6	Biochemie III
**Y287**	YMJH1-1a	MATa; *his3-1*; *leu2-0*; *met15-0*; *ura3-0*; KANMX::GAL::HA-*PWP2*	This study
**Y289**	YCMS3-1a	MATa; *his3-1*; *leu2-0*; *met15-0*; *ura3-0*; KANMX::GAL::HA-*PWP2*; *YLR409C*-TAP::*HIS3*MX6	This Study
**Y339**	YCMS4-1a	MATa; *his3-1*; *leu2-0*; *met15-0*; *ura3-0*; KANMX::GAL::HA-*PWP2*; *YLR409C*-GFP::*HIS3*MX6	This Study
**Y344**	YCMS5-1a	MATa; *his3-1*; *leu2-0*; *met15-0*; *ura3-0*; KANMX::GAL::HA-*PWP2*; *YLR409C*-GFP::*HIS3*MX6; *RPA135*-mCHERRY::Hph	This Study

Figure Legend

<u>Figure S1:</u> General SSU-processome proteins.

As indicated in figure 1, Utp21-TAP-purified proteins were identified and quantitated by Mass Spectrometry. Isobaric labelling of peptides (iTRAQ, see Materials and Methods) was used to compare the protein levels in wild-type Utp21 and in Pwp2 deleted strains. The bar graphs represent the ratios for the indicated purifications represented in logarithmic scale. The average of ratios for proteins identified in tree experiments with at least two peptides is shown for SSU-processome factors that do not fit in the categories shown in figure 1D, 1E and 1F.

<u>Figure S2:</u> Sequence conservation and electrostatic surface potential of Pwp2p tandem repeat.

Pwp2p tandem repeat structure is displayed in **A)** under the same orientation as Figure 1A. The residue conservation has been calculated using the sequence alignment displayed in Figure S1 and using the CONSURF server interface [7](Panel A, C, E, G). Only residues conserved above 80 % are displayed in a gradient from orange (80 % conservation) to purple (identical). Panels B, D, F, and H display the

electrostatics surface potential as calculated from PyMol using APBS plugin [8](blue = positively charged, red = negatively charged). Panels C-D; E-F, and G-H were generated from the orientation A-B by iterative rotations of 90° in the x-axis as indicated. Some conserved residues are indicated, mutated residues used in this study are shown in bold.

<u>Figure S3:</u> Sequence alignments of Pwp2 orthologues.

Sequences were aligned using ClustalX (6) and displayed using Jalview (7). Disordered regions of Pwp2 are boxed. Numbering on top of the sequences refers to the yeast Pwp2 while numbering beneath the alignment is an absolute count. The secondary structures indicated above the sequences are deduced from the crystal structure of Pwp2p (this work) and inferred from the crystal structure of *C.t* Utp1 [9].* indicates the amino acids substituted by site directed mutagenesis. Other relevant sequences for this work are also indicated.

<u>Figure S4:</u> Growth assays and expression analysis of different *pwp2* mutants.

A-B) Drop assays of Pwp2 mutant strains. The yCMS3 strain was transformed with the backbone plasmid YCplac33 (“empty”), the wild-type *PWP2* containing plasmid pJPF39 (“*PWP2*”) or a plasmid containing a mutation in *PWP2*. Serial dilutions of cells were plated either on SCG medium allowing for the expression of a genomic copy of an HA-tagged copy of PWP2, or in the SCD medium where only the Pwp2 copy encoded in the plasmid is expressed. Incubation temperature is indicated at the right side. **C)** Expression analysis of Pwp2 mutants. 5 ODs of cell cultures expressing the different Pwp2p mutants were obtained and proteins were obtained by TCA extraction. Samples were resolved in 8% SDS-PAGE for western blot analysis with antibodies against GFP, Pwp2p and Utp18p. **D)** Doubling time of different pwp2 mutants at 30° and 37°C. Expression of the genomic copy of PWP2 (g*PWP2* “ON”) allows a similar doubling time for all strains regardless of the *PWP2* allele co-expressed at both temperatures. In contrast, *pwp2-ΔC66* mutant show a mild growth defect at both temperatures in absence of the endogenous Pwp2p (g*PWP2* “OFF”) and *pwp2-K613E/R614E* mutant shows a mild growth defect only at 37°C.

<u>Figure S5:</u> Relevance of the CTD domain of Pwp2 for cell growth and trans-complementation analysis between tWD and CTD domains of Pwp2.

A) Drop assay of Pwp2 mutant strains. The yCMS3 strain was transformed with the backbone plasmid YCplac33 ("empty"), the wild-type *PWP2* containing plasmid pJPF39 ("*PWP2*") or a plasmid containing a mutation in *PWP2*. Serial dilutions of cells were plated either on SCG medium allowing for the expression of a genomic copy of an HA-tagged copy of PWP2, or in the SCD medium where only the Pwp2 copy encoded in the plasmid is expressed. Incubation temperature is indicated at the right side. **B)** Doubling time of the Pwp2p tWD/CTD*Δ66* mutant shows a stronger growth defect at 20 °C when compared to a strain expressing the wt Pwp2p. **C)** Trans-complementation assay. The yCMS3 strain was co-transformed with two different plasmids. On one hand, the backbone plasmid YCplac33 ("empty"), the plasmid containing the wild-type *PWP2* ("*PWP2*") or a plasmid containing one of the *Pwp2p* truncated forms (*pwp2-ΔC103*, *pwp2-ΔC206*). On the other hand, a plasmid containing the coding region of the C-terminal fragments of Pwp2 complementary to the Pwp2p-ΔC206 or Pwp2p-ΔC103, CTD_{232} or CTD117 respectively (see Fig. 3A). Expression of the CTD of Pwp2p is repressed in presence of Doxycyclin (1mg/ml). All clones grew similar on SCG medium allowing for the "overexpression" of a genomic copy of an HA-tagged copy of PWP2 (g*PWP2* "ON") and lacking Doxycyclin (CTD "ON"). In contrast, the expression of the two complementary coding regions of Pwp2p correlates with a reduction in the doubling time (compare CTD "ON" and "OFF" in absence of endogenous Pwp2p).

<u>Figure S6:</u> UTP-B subunits overexpression in bacteria.

A) Schematic representation of Utp21p, Utp12p, and Utp13p domain organization. Interactions observed in Figure 3E and 3F are summarized. **B-D)** In order to ensure that proteins were expressed in bacteria an assay was set-up in which the total bacterial cell content is analysed before and after induction by IPTG and revealed on SDS-PAGE and Coomassie-Blue staining. Overexpressed proteins are identified with an arrow.

<u>Figure S7:</u> Utp12p(688-943)-Utp13p(648-817) complex purification.

The minimal core domain necessary for Utp12p and Utp13p interaction was expressed and purify to

homogeneity. The size exclusion chromatography column profile and the corresponding SDS-PAGE analysis are shown. Additionally, a SDS-PAGE analysis displays the complex prior and after TEV cleavage.

Figure S8: Localization of Utp21p and expression level of the Utp21p-GFP.

The strain yCMS4-1a (containing *GAL::HA-PWP2* and *UTP21-GFP* at the endogenous *loci*) was transformed with plasmids containing the Pwp2p wild type allele or alleles encoding the truncated forms of Pwp2p (*pwp2-ΔC32*, p*wp2-ΔC66* or p*wp2-ΔC103*). Exponentially growing cells were shifted from galactose to glucose containing medium and further cultured for 14 h (Wt, *pwp2-ΔC32* and *pwp2-ΔC66*) or 10 h (*pwp2-ΔC103*) keeping exponential growth conditions. In both cases cells were harvested and processed for fluorescent microscopy analysis as indicated in the Material and Methods. From left to right: DAPI staining (blue), Utp21p:GFP (green) and merged images. The scale bar represents 4 μm.

1 Gietz, R. D. & Sugino, A. New yeast-Escherichia coli shuttle vectors constructed with in vitro mutagenized yeast genes lacking six-base pair restriction sites. *Gene* **74**, 527-534 (1988).

2 Janke, C. *et al.* A versatile toolbox for PCR-based tagging of yeast genes: new fluorescent proteins, more markers and promoter substitution cassettes. *Yeast* **21**, 947-962, doi:10.1002/yea.1142 (2004).

3 Belli, G., Gari, E., Piedrafita, L., Aldea, M. & Herrero, E. An activator/repressor dual system allows tight tetracycline-regulated gene expression in budding yeast. *Nucleic Acids Res* **26**, 942-947 (1998).

4 Gari, E., Piedrafita, L., Aldea, M. & Herrero, E. A set of vectors with a tetracycline-regulatable promoter system for modulated gene expression in Saccharomyces cerevisiae. *Yeast* **13**, 837-848, doi:10.1002/(SICI)1097-0061(199707)13:9<837::AID-YEA145>3.0.CO;2-T (1997).

5 Longtine, M. S. *et al.* Additional modules for versatile and economical PCR-based gene deletion and modification in Saccharomyces cerevisiae. *Yeast* **14**, 953-961, doi:10.1002/(SICI)1097-0061(199807)14:10<953::AID-YEA293>3.0.CO;2-U (1998).

6 Diebold, M. L., Fribourg, S., Koch, M., Metzger, T. & Romier, C. Deciphering correct strategies for multiprotein complex assembly by co-expression: application to complexes as large as the histone octamer. *J Struct Biol* **175**, 178-188, doi:10.1016/j.jsb.2011.02.001 (2011).

7 Ashkenazy, H., Erez, E., Martz, E., Pupko, T. & Ben-Tal, N. ConSurf 2010: calculating evolutionary conservation in sequence and structure of proteins and nucleic acids. *Nucleic Acids Res* **38**, W529-533, doi:10.1093/nar/gkq399 (2010).

8 Baker, N. A., Sept, D., Joseph, S., Holst, M. J. & McCammon, J. A. Electrostatics of nanosystems: application to microtubules and the ribosome. *Proc Natl Acad Sci U S A* **98**, 10037-10041, doi:10.1073/pnas.181342398 (2001).

9 Zhang, C. *et al.* Integrative structural analysis of the UTPB complex, an early assembly factor for eukaryotic small ribosomal subunits. *Nucleic Acids Res* **44**, 7475-7486, doi:10.1093/nar/gkw562 (2016).

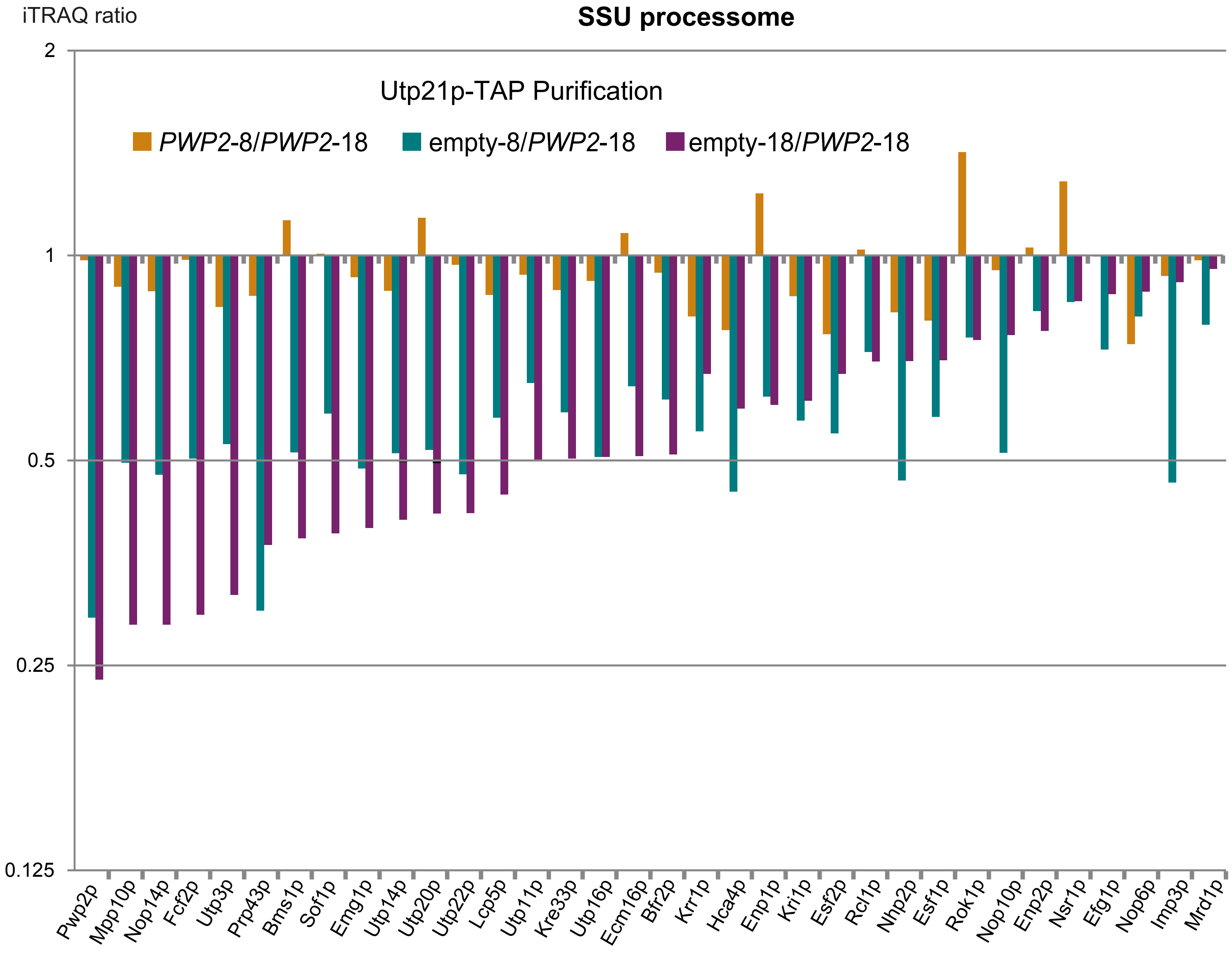

iTRAQ ratio
SSU processome
Utp21p-TAP Purification
PWP2-8/PWP2-18
empty-8/PWP2-18
empty-18/PWP2-18
2
1
0.5
0.25
0.125
Pwp2p
Mpp10p
Nop14p
Fcf2p
Utp3p
Prp43p
Bms1p
Sof1p
Emg1p
Utp14p
Utp20p
Utp22p
Lcp5p
Utp11p
Kre33p
Utp16p
Ecm16p
Bfr2p
Krr1p
Hca4p
Enp1p
Kri1p
Esf2p
Rcl1p
Nhp2p
Esf1p
Rok1p
Nop10p
Enp2p
Nsr1p
Efg1p
Nop6p
Imp3p
Mrd1p

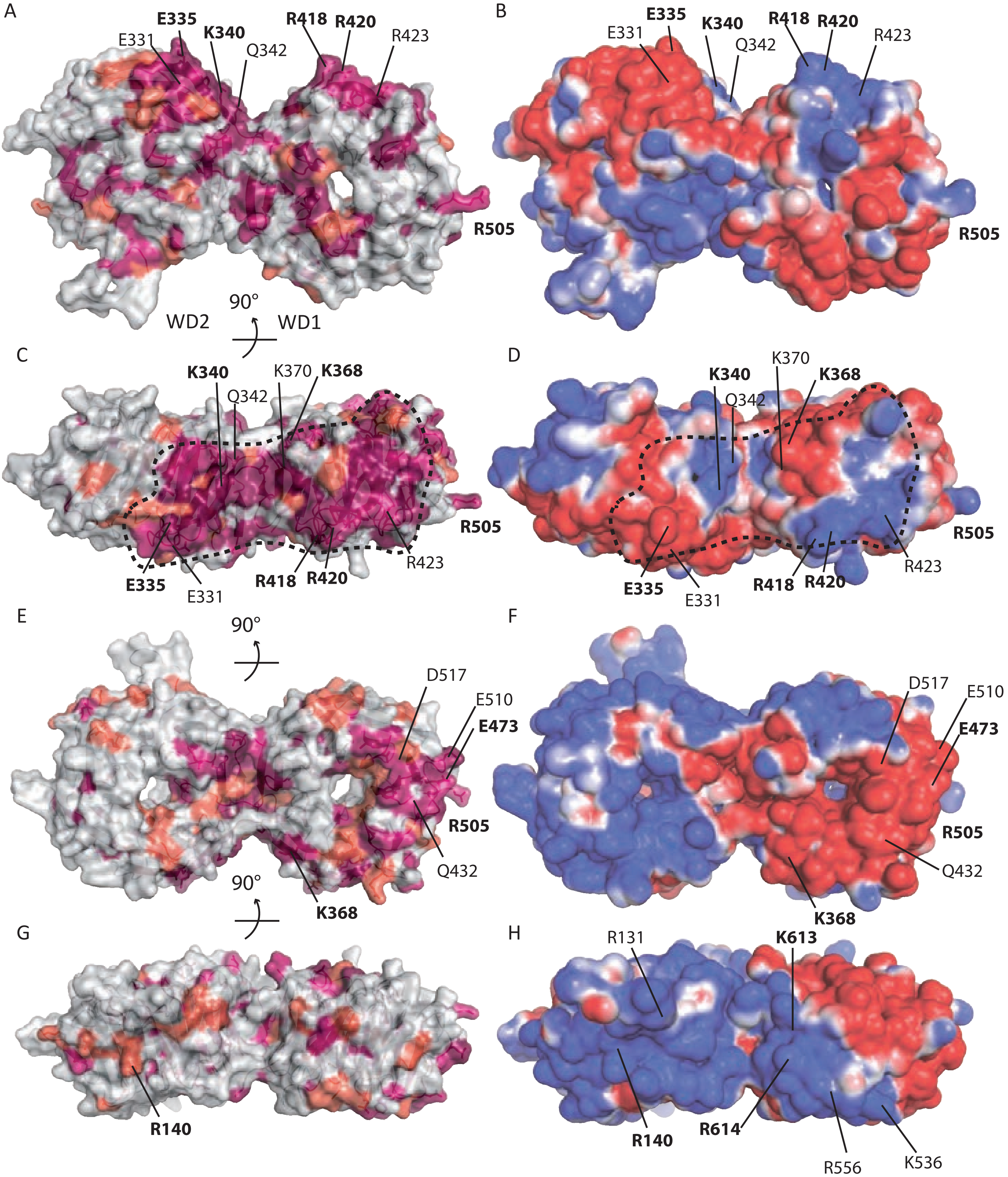
A
E335
K340
R418
R420
E331
Q342
R423
R505
90°
WD2
WD1
B
E335
K340
R418
R420
E331
Q342
R423
R505
C
K340
K370
K368
Q342
R505
E335
E331
R418
R420
R423
D
K370
K340
K368
Q342
R505
E335
E331
R418
R420
R423
E
90°
D517
E510
E473
R505
Q432
90°
K368
F
D517
E510
E473
R505
Q432
K368
G
R140
H
R131
K613
R140
R614
R556
K536

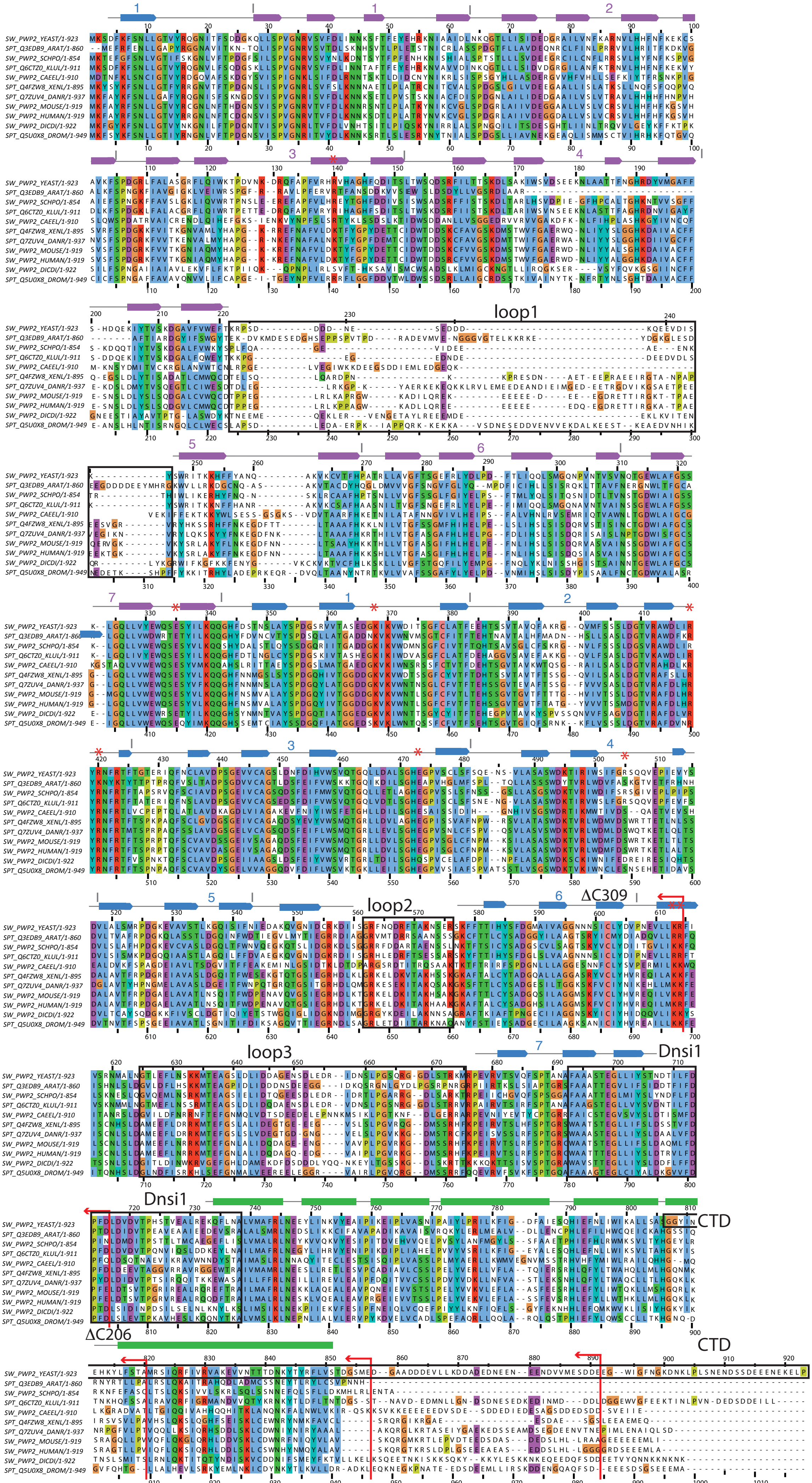
SW_PWP2_YEAST/1-923
SPT_Q3EDB9_ARAT/1-860
SPT_PWP2_SCHPO/1-854
SPT_Q6CTZ0_KLUL/1-911
SPT_PWP2_CAEEL/1-910
SPT_Q4FZW8_XENL/1-895
SPT_Q7ZUV4_DANR/1-937
SPT_PWP2_MOUSE/1-919
SPT_PWP2_HUMAN/1-919
SPT_PWP2_DICDI/1-922
SPT_Q5U0X8_DROM/1-949
loop1
loop2
loop3
Dnsi1
CTD
ΔC309
ΔC206
ΔC103
ΔC66
ΔC32

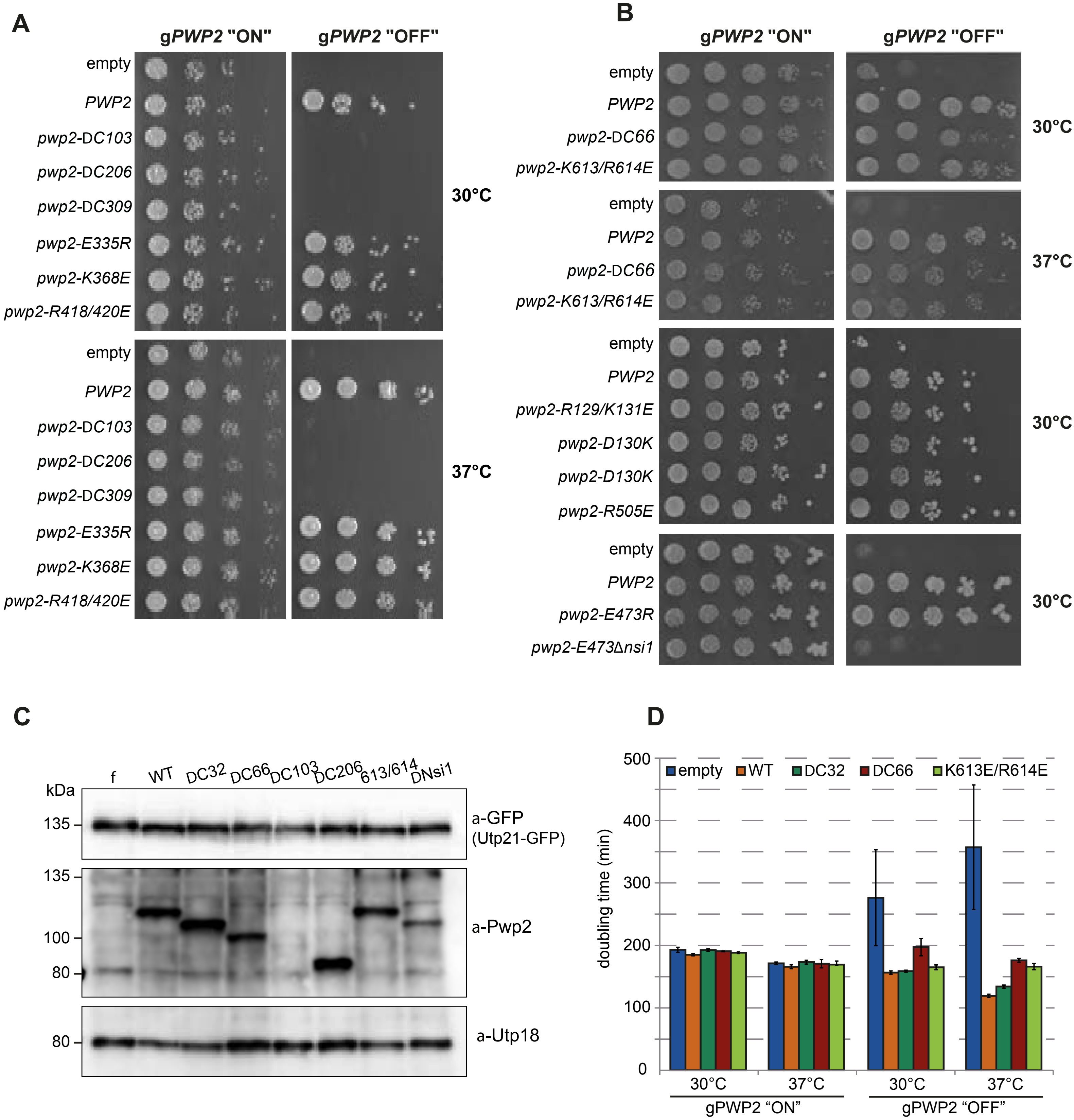
A
gPWP2 "ON"
gPWP2 "OFF"
empty
PWP2
pwp2-DC103
pwp2-DC206
pwp2-DC309
pwp2-E335R
pwp2-K368E
pwp2-R418/420E
30°C
37°C
B
pwp2-DC66
pwp2-K613/R614E
pwp2-R129/K131E
pwp2-D130K
pwp2-R505E
pwp2-E473R
pwp2-E473Δnsi1
C
f
WT
DC32
DC66
DC103
DC206
613/614
DNsi1
kDa
135
100
80
a-GFP
(Utp21-GFP)
a-Pwp2
a-Utp18
D
empty
WT
DC32
DC66
K613E/R614E
doubling time (min)
0
100
200
300
400
500
gPWP2 "ON"
gPWP2 "OFF"

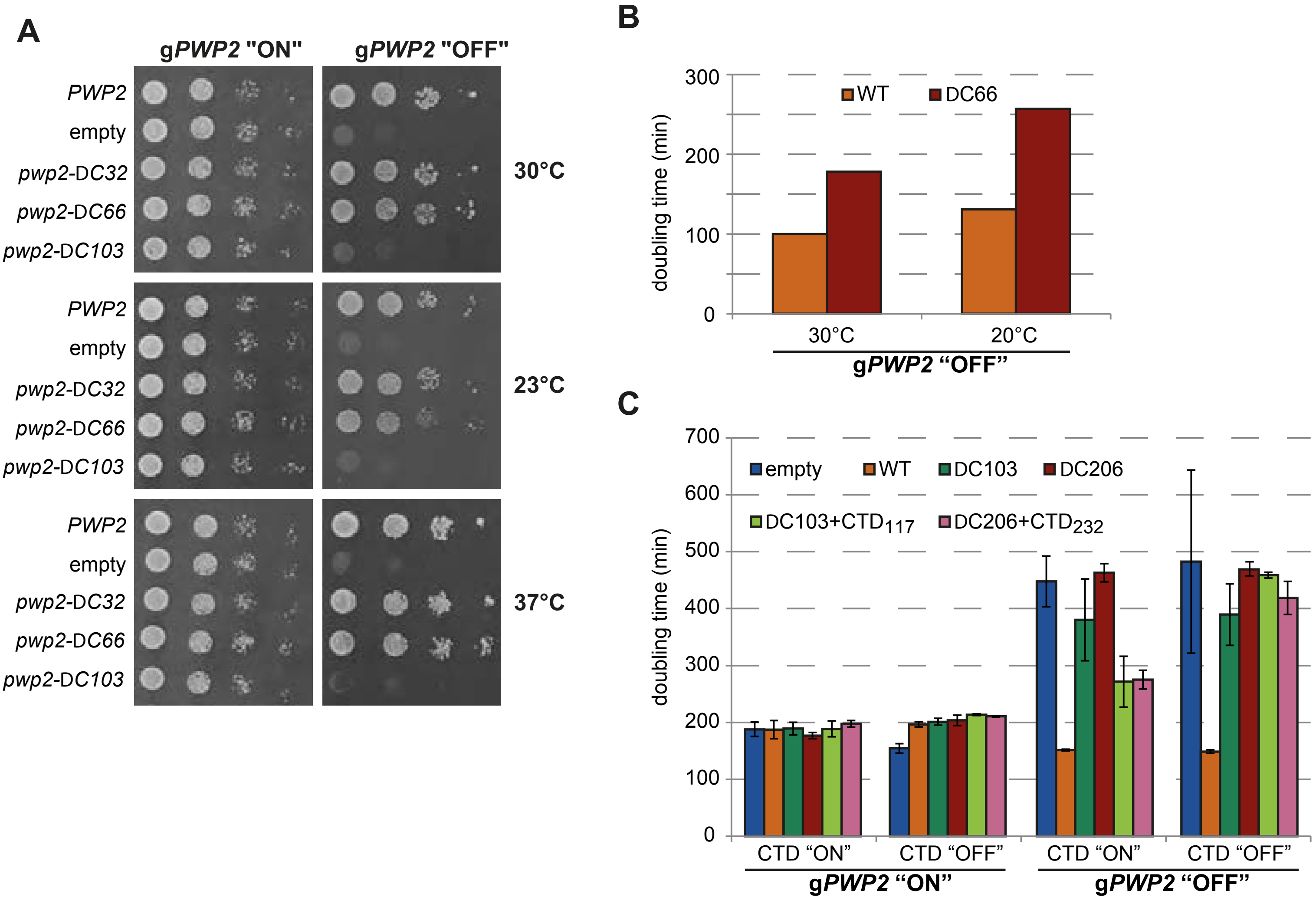

A
gPWP2 "ON"
gPWP2 "OFF"
PWP2
empty
pwp2-DC32
pwp2-DC66
pwp2-DC103
30°C
23°C
37°C
B
300
200
100
0
WT
DC66
doubling time (min)
30°C
20°C
gPWP2 "OFF"
C
700
600
500
400
300
200
100
0
empty
WT
DC103
DC206
DC103+CTD117
DC206+CTD232
doubling time (min)
CTD "ON"
CTD "OFF"
CTD "ON"
CTD "OFF"
gPWP2 "ON"
gPWP2 "OFF"

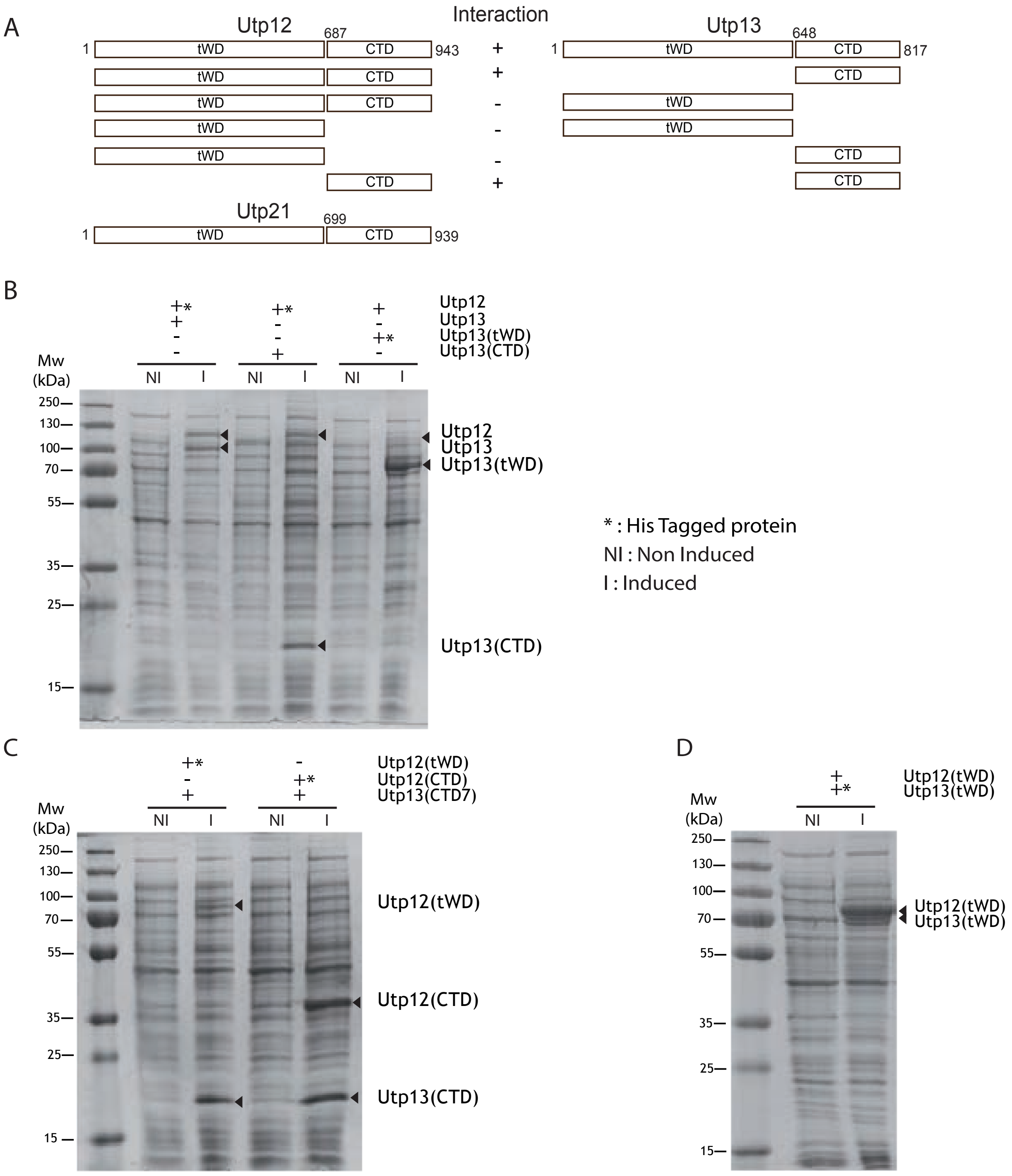
A
Interaction
Utp12
687
1
tWD
CTD
943
+
Utp13
648
1
tWD
CTD
817
+
-
-
-
+
Utp21
699
1
tWD
CTD
939
B
Utp12
Utp13
Utp13(tWD)
Utp13(CTD)
Mw
(kDa)
NI
I
250
130
100
70
55
35
25
15
Utp12
Utp13
Utp13(tWD)
Utp13(CTD)
* : His Tagged protein
NI : Non Induced
I : Induced
C
Utp12(tWD)
Utp12(CTD)
Utp13(CTD7)
Mw
(kDa)
NI
I
Utp12(tWD)
Utp12(CTD)
Utp13(CTD)
D
Utp12(tWD)
Utp13(tWD)
Mw
(kDa)
NI
I
Utp12(tWD)
Utp13(tWD)

Size exclusion chromatography
of Utp12(CTD) and Utp13(CTD)

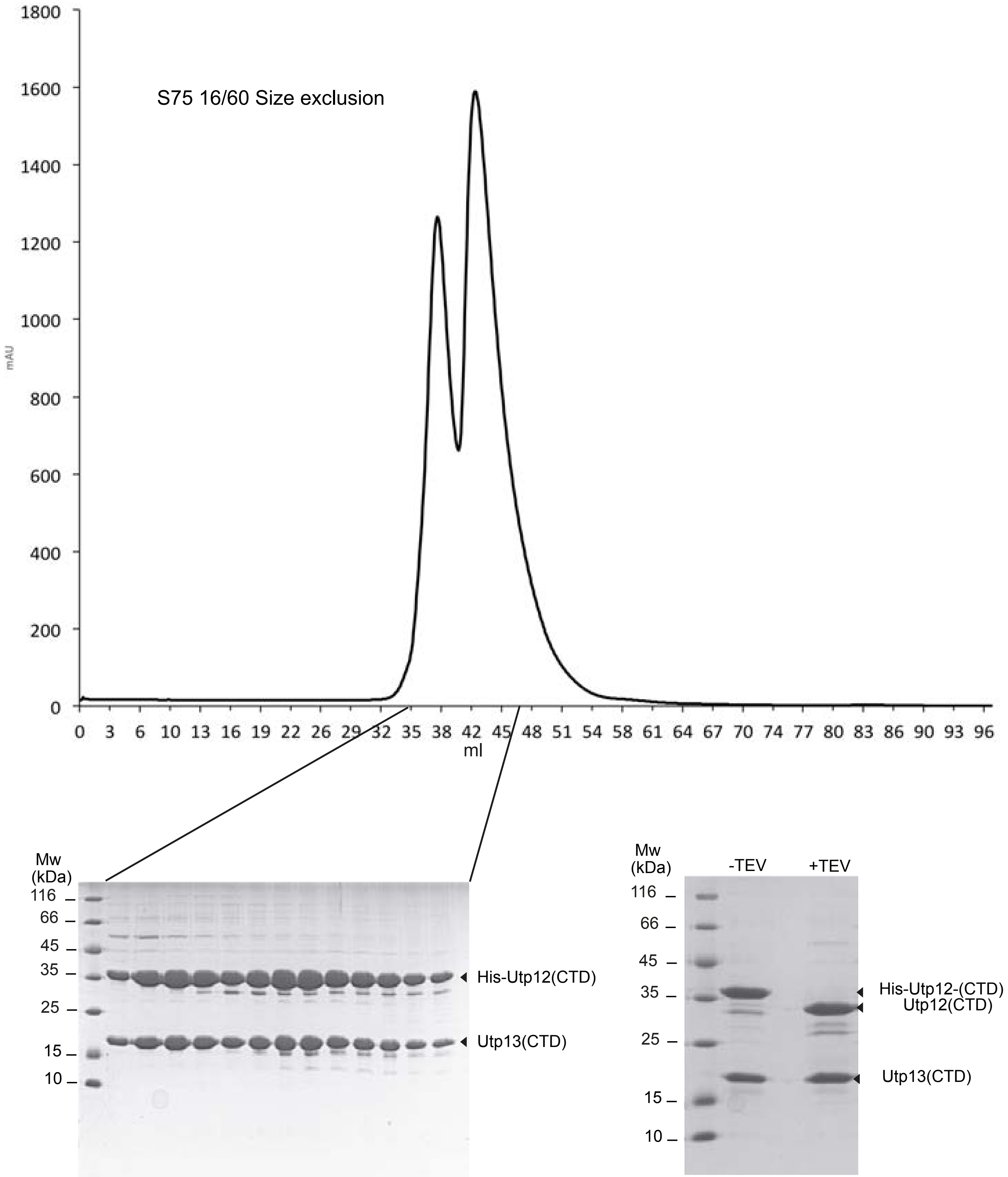

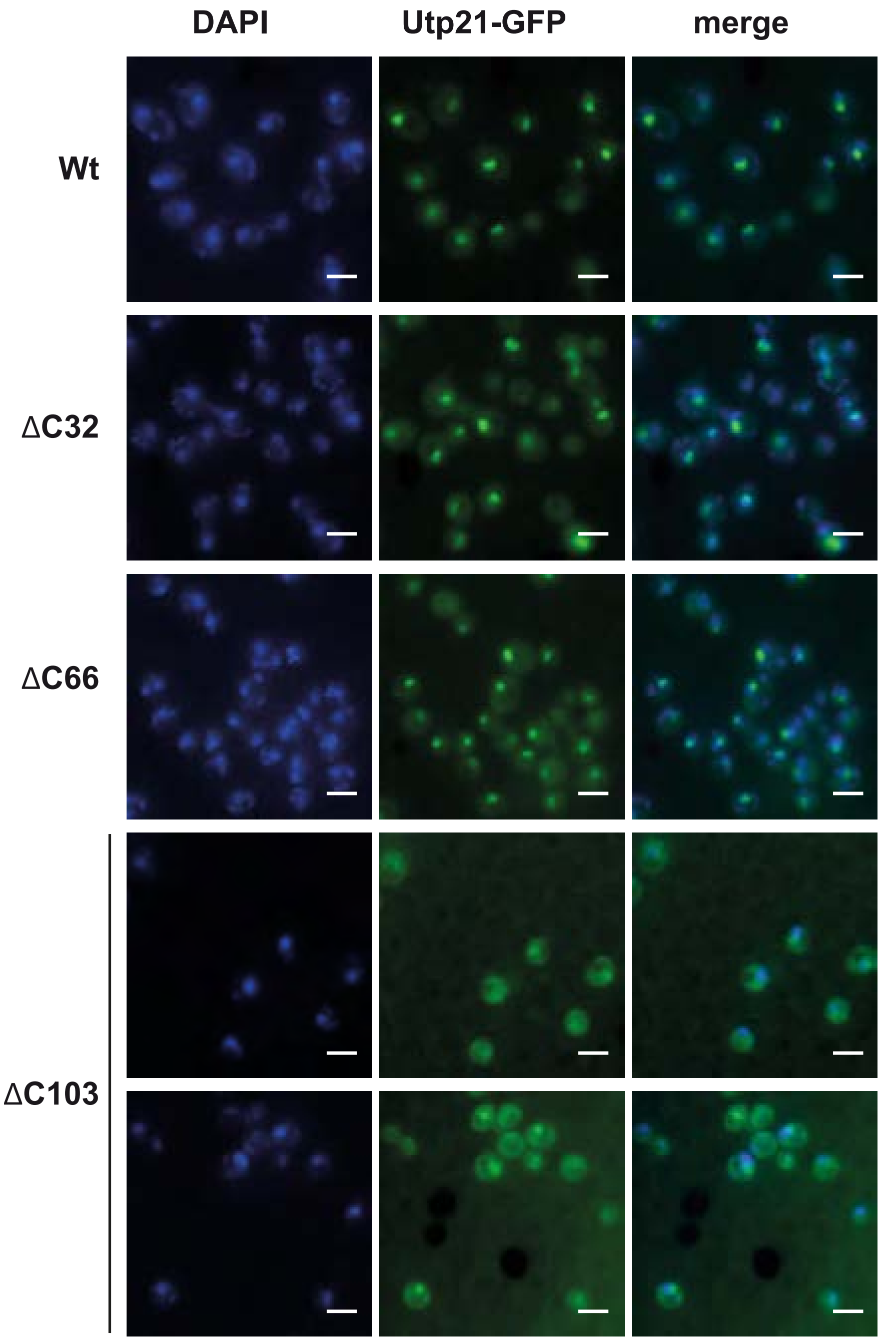
DAPI
Utp21-GFP
merge
Wt
ΔC32
ΔC66
ΔC103
